# How to Tackle Phylogenetic Discordance in Recent and Rapidly Radiating Groups? Developing a Workflow Using *Loricaria* (Asteraceae) as an Example

**DOI:** 10.3389/fpls.2021.765719

**Published:** 2022-01-07

**Authors:** Martha Kandziora, Petr Sklenář, Filip Kolář, Roswitha Schmickl

**Affiliations:** ^1^Department of Botany, Faculty of Science, Charles University, Prague, Czechia; ^2^Institute of Botany, The Czech Academy of Sciences, Průhonice, Czechia

**Keywords:** rapid radiation, hybridization, workflow, incomplete lineage sorting, gene tree discordance, cytonuclear discordance

## Abstract

A major challenge in phylogenetics and -genomics is to resolve young rapidly radiating groups. The fast succession of species increases the probability of incomplete lineage sorting (ILS), and different topologies of the gene trees are expected, leading to gene tree discordance, i.e., not all gene trees represent the species tree. Phylogenetic discordance is common in phylogenomic datasets, and apart from ILS, additional sources include hybridization, whole-genome duplication, and methodological artifacts. Despite a high degree of gene tree discordance, species trees are often well supported and the sources of discordance are not further addressed in phylogenomic studies, which can eventually lead to incorrect phylogenetic hypotheses, especially in rapidly radiating groups. We chose the high-Andean Asteraceae genus *Loricaria* to shed light on the potential sources of phylogenetic discordance and generated a phylogenetic hypothesis. By accounting for paralogy during gene tree inference, we generated a species tree based on hundreds of nuclear loci, using Hyb-Seq, and a plastome phylogeny obtained from off-target reads during target enrichment. We observed a high degree of gene tree discordance, which we found implausible at first sight, because the genus did not show evidence of hybridization in previous studies. We used various phylogenomic analyses (trees and networks) as well as the D-statistics to test for ILS and hybridization, which we developed into a workflow on how to tackle phylogenetic discordance in recent radiations. We found strong evidence for ILS and hybridization within the genus *Loricaria*. Low genetic differentiation was evident between species located in different Andean cordilleras, which could be indicative of substantial introgression between populations, promoted during Pleistocene glaciations, when alpine habitats shifted creating opportunities for secondary contact and hybridization.

## Introduction

While rapidly radiating groups are interesting to science due to their potential to understand evolution, adaptation, and the impact of environmental change on biodiversity, they pose one of the biggest challenges in resolving the tree of life (Whitfield and Lockhart, 2007; Song et al., 2012; Escudero et al., 2020; Morales-Briones et al., 2021). Phylogenies of rapid radiations have short internal branches due to the fast succession of species. This rapid accumulation of species can be the result of different and non-exclusive processes, such as geographic isolation, sexual selection or ecological adaptation (Schluter, 2000; Givnish, 2015). The short time between speciation events in rapid radiations increases the probability of incomplete lineage sorting (ILS), i.e., the phenomenon of ancestral polymorphism persisting between successive speciation events (Maddison, 1997). This potentially reduces phylogenetic signal (Townsend, 2007; Whitfield and Lockhart, 2007), as the different topologies of the gene trees expected under ILS lead to gene tree discordance, i.e., not all gene trees represent the species tree. The advent of phylogenomics has not only brought novel methods of generating large datasets, but also new methods of inferring phylogenetic trees and networks. Several available methods to reconstruct the species tree and phylogenetic networks account for ILS (e.g., Than et al., 2008; Vachaspati and Warnow, 2015; Solís-Lemus et al., 2017; Zhang et al., 2017). ILS can be addressed by using multi-species coalescent (MSC) methods for phylogenetic reconstruction, where the different evolutionary histories of loci are considered. Especially in lineages that show a high degree of ILS, species tree estimations are usually more reliable than concatenation (Jiang et al., 2020).

Besides ILS, other processes can lead to phylogenetic discordance, both among gene trees (hereafter referred to as gene tree discordance) and across genomes (among different genomic compartments within a genome; hereafter referred to as cytonuclear discordance). Within plants these processes are mainly hybridization and whole-genome duplication (WGD; Degnan and Rosenberg, 2009). Hybridization frequently occurs under the form of ‘introgressive hybridization,’ i.e., the introduction of syntenic nucleotide variation from a donor species into the genome of a recipient species, by means of hybridization and backcrossing (Anderson and Hubricht, 1938). Evidence for hybridization from phylogenetic datasets has traditionally been obtained from cytonuclear discordance (i.e., the incongruence between nuclear and plastome trees) and using graph-based networks (e.g., NeighborNet, SuperQ; Bryant and Moulton, 2004; Grunewald et al., 2013). While these approaches remain applicable in the phylogenomics era, they rather depict reticulation; commonly interpreted as evidence for hybridization, however this is non-exclusive (Huson and Bryant, 2006; Degnan, 2018). In contrast, a few methods that account for ILS and simultaneously address hybridization exist which allow testing of hybrid origin, among them the D-statistics (ABBA-BABA statistics; Patterson et al., 2012) and phylogenetic networks (Than et al., 2008; Solís-Lemus et al., 2017). As these model-based approaches are computationally demanding and feasible only for a small number of samples and putative hybridization events (Kamneva et al., 2017; Folk et al., 2018), testing for hybrid origins in phylogenomic datasets remains a challenge.

Hybrids are frequently meiotically stabilized via WGD, but WGD events also occur in the absence of hybridization. After a WGD event, gene copies are subsequently lost (e.g., Xiang et al., 2017). If duplicated non-homologous sequences are not differentiated into their orthologous pairs, the orthology assumption for phylogenetic reconstruction is violated. Alignments that consist of paralogous sequences may lead to biased phylogenetic inference (Fitch, 1970; Gabaldón, 2008; Yang and Smith, 2014) or not (Yan et al., 2021). The best practice to account for paralogy in phylogenetic reconstruction is under debate. Four strategies can be followed: (1) deleting paralogous loci from the analysis (Jones et al., 2019; Larridon et al., 2020); (2) retrieving both ortho- and paralogous copies of the loci without separating them into different alignments and proceeding with a gene duplication-aware species tree method (Zhang et al., 2020a); (3) retrieving both ortho- and paralogous copies without separating them into different alignments and proceeding with an ILS-aware species tree method (Yan et al., 2021); and (4) retrieving all copies, both ortho- and paralogous, and creating orthologous alignments, from which gene trees are inferred, before building the species tree (Gizaw et al., 2021; this study).

Once gene tree discordance is found in a dataset, its sources should be deciphered, as it can lead to wrong estimations of phylogenetic relationships (Huson and Bryant, 2006). Apart from the evolutionary processes mentioned above, methodological artifacts due to missing data, scarce sampling of taxa, and incorrect model specifications can be additional sources of gene tree discordance (Molloy and Warnow, 2018; Nute et al., 2018). Especially when resolving phylogenetic relationships in rapid radiations, the degree of gene tree discordance is expected to be high, as a large number of loci has to be employed, which increases the likelihood of sampling loci that evolved under ILS, hybridization, and WGD (Degnan and Rosenberg, 2009). As such, rapidly radiating groups present a challenge in terms of resolution in phylogenomic datasets, at least when using currently available computational methods (Esselstyn et al., 2017; Reddy et al., 2017). Studies about the sources of genome and gene tree discordance either focus on evolutionary model organisms, where *a priori* knowledge about putative hybridization events is larger (e.g., Meier et al., 2017; Lee-Yaw et al., 2019) or ancient radiations, where the effect of ILS is decreased due to species extinction (e.g., Rosidae, Sun et al., 2015; Amaranthaceae s.l., Morales-Briones et al., 2021). In contrast, phylogenetic discordance in young radiating groups only recently gained attention: *Lachemilla* Focke (Rydb.) (Morales-Briones et al., 2018), *Lomatium* Raf. (Ottenlips et al., 2021), and *Veronica* L. (Thomas et al., 2021). A lack of data sources, such as phylogenies based on Sanger sequence markers, detailed morphological evaluations, and flow cytometric measurements in combination with chromosome counts, make it particularly difficult to disentangle sources of phylogenetic incongruence in young understudied groups.

Rapidly radiating groups can be found in young biodiversity hotspots, such as the high altitude areas of tropical South America (Madriñán et al., 2013). Comprehensive sampling of lineages that are either large in species number, cover large geographic areas or include many micro-endemics often pose substantial taxonomic and fieldwork challenges. In the past, the use of a few Sanger sequence markers or the plastome often did not provide sufficient resolution at shallow phylogenetic levels. As such, many of these lineages or genera are understudied and remain poorly understood, including several Andean radiations: e.g., *Astragalus* L. (Bagheri et al., 2017); *Diplostephium* Kunth (Vargas et al., 2017); Espeletiinae (Diazgranados and Barber, 2017; Cortés et al., 2018); *Lupinus* L. (Drummond et al., 2012; Contreras-Ortiz et al., 2018); and *Senecio* L. (Kandziora et al., 2016).

The family Asteraceae is one of the youngest and most species-rich families among the angiosperms and accounts for a large diversity within tropical alpine ecosystems (Sklenář et al., 2011; Panero and Crozier, 2016). WGD events and hybridization are common for many members of the Asteraceae (Smissen et al., 2011; Galbany-Casals et al., 2014; Barker et al., 2016; Huang et al., 2016; Zhang et al., 2021). For this study, we chose the high-Andean genus *Loricaria* Wedd. from the tribe Gnaphalieae as a representative of a young radiating group. The genus comprises 19 species and has an estimated stem age of 6 million years (Ma; crown age of 4 Ma) according to Nie et al. (2016). The genus occurs above 3500m in the tropical Andes from Bolivia to Colombia. During Pleistocene glacial cycles, the tropical alpine ecosystem shifted downwards in the cold and drier periods (Van der Hammen, 1985; Hooghiemstra and Van der Hammen, 2004; Flantua et al., 2019) and species changed their ranges, met, and potentially hybridized. Interestingly, there was no evidence for hybridization in *Loricaria* based on amplified fragment length polymorphism (AFLP) data, and polyploids have not been detected to date (Kolář et al., 2016).

In this study, we addressed phylogenetic relationships among *Loricaria* using Hyb-Seq (Weitemier et al., 2014). We encountered a high degree of discordance, both among gene trees and across genomes, which seems to be common for Asteraceae (Siniscalchi et al., 2021). We then aimed to disentangle ILS, hybridization, and WGD as possible sources of the immense gene tree discordance, and we established a workflow for phylogenetic inference of young radiating groups that accounts for these sources of discordance. We additionally accounted for a possible impact of missing data on gene tree discordance. Our workflow is especially useful for non-model groups, for which often only limited knowledge exists about hybridization events and polyploidy.

## Materials and Methods

### Taxonomic Focus

*Loricaria* is a genus restricted to the high elevation habitats of the tropical Andes. The genus comprises small dioecious shrubs with scale-like leaves, which is a morphological convergence to certain gymnosperm genera (Cuatrecasas, 1954). Currently, 19 species are accepted, 17 as a result of the synopsis of the genus by Cuatrecasas (1954), and two species described by Dillon and Sagastegui Alva (1986), and Hind (2004). Morphological investigations of herbarium specimens plus signatures of potential cryptic speciation within *L. thuyoides* (Lam.) Sch. Bip. (Kolář et al., 2016) suggested that there is a substantial degree of taxonomic uncertainty and that there potentially exist more species than are described to date.

The genus has been divided into three different sections (Table 1), primarily based on the position of the flower heads, which is axillary in sect. *Thyopsis* and sect. *Graveoleum* and terminal in sect. *Terminalia*. Section *Graveoleum* is differentiated from the other two sections by a glandulose-pilose ovary and glandulose-pubescent leaves. *Loricaria graveolens* (Sch. Bip.) Wedd. is the only member of sect. *Graveoleum*. Distribution information and section assignation are taken from Cuatrecasas (1954), Dillon and Sagastegui Alva (1986), and Hind (2004).

**TABLE 1:** Species and characteristics of *Loricaria*.

Species	Section	Clade	Capitulum position	Distribution
*L. graveolens* (Sch. Bip.) Wedd.	Graveoleum	Graveolens	Axilliary	Peru (Cuatrecasas, 1954)
*L. ollgaardii* M.O. Dillon & Sagast.	Thyopsis	Unknown	Terminal	Ecuador (Dillon and Sagastegui Alva, 1986)
*L. complanata* (Sch. Bip.) Wedd.	Thyopsis	Axilliary	Axilliary	Ecuador, Colombia (Cuatrecasas, 1954)
*L. thuyoides* (Lam.) Sch. Bip.	Thyopsis	Axilliary	Axilliary	Peru, Ecuador, Colombia (Cuatrecasas, 1954)
*L. scolopendra* (Hook.) Kuntze	Thyopsis	Axilliary	Axilliary	Ecuador (Cuatrecasas, 1954)
*L. pauciflora* Cuatrec.	Thyopsis	Axilliary	Axilliary	Ecuador (Cuatrecasas, 1954)
*L. azuayensis* Cuatrec.	Thyopsis	Axilliary	Axilliary	Ecuador (Cuatrecasas, 1954)
*L. cinerea* D. J. N. Hind	Thyopsis	unknown	Axilliary and terminal	Ecuador (Hind, 2004)
*L. lagunillensis* Cuatrec.	Thyopsis	unknown	Axilliary	Colombia (Cuatrecasas, 1954)
***L. leptothamna* (Mattf.) Cuatr.**	**Thyopsis**	**(Terminal)**	**Terminal**	Peru (Cuatrecasas, 1954)
*L. puracensis* Cuatrec.	Terminalia	Terminal	Terminal	Colombia (Cuatrecasas, 1954)
*L. lucida* Cuatrec.	Terminalia	Unknown	Terminal	Peru (Cuatrecasas, 1954)
*L. ferruginea* (Ruiz & Pav.) Wedd.	Terminalia	Terminal	Terminal	Peru, Ecuador (Cuatrecasas, 1954)
*L. lycopodinae* Cuatrec.	Terminalia	Terminal	Terminal	Peru (Cuatrecasas, 1954)
*L. antisanensis* Cuatrec.	Terminalia	Terminal	Terminal	Ecuador (Cuatrecasas, 1954)
*L. ilinissae* (Benth.) Cuatrec.	Terminalia	Terminal	Terminal	Ecuador (Cuatrecasas, 1954)
*L. macbridei* Cuatrec.	Terminalia	Unknown	Terminal	Peru (Cuatrecasas, 1954)
***L. colombiana* Cuatrec.**	**Terminalia**	**Terminal**	**Axilliary**	Colombia (Cuatrecasas, 1954)
***L. unduaviensis* Cuatrec.**	**Thyopsis**	**Terminal**	**(Axilliary) and terminal**	Bolivia (Cuatrecasas, 1954)

*In bold are highlighted where the species’ sectional assignment does not match their placement in clades and/or position of the capitula.*

The genus belongs to the tribe Gnaphalieae, which has its species richness concentrated in the southern hemisphere (Nie et al., 2016). Hybridization has been inferred for this tribe (Galbany-Casals et al., 2014; Barker et al., 2016), thus phylogenetic discordance can be expected. Further, within the tribe Gnaphalieae the most recent common ancestor (mrca) of the FLAG-clade, which includes *Loricaria* [defined in Galbany-Casals et al. (2010), the acronym stands for the species-rich genera within this clade: *Filago* L., *Leontopodium* R. Br. ex Cass., *Antennaria* Gaertn., and *Gamochaeta* Wedd.], likely underwent a hybridization plus WGD event (Smissen et al., 2011). Further, the Gnaphalieae experienced a WGD event about 10 Ma ago (Huang et al., 2016; Zhang et al., 2020b). These WGD events are expected to add phylogenetic discordance.

### Sampling and DNA Sequencing

Sampling was carried out over a period of 12 years (2006–2018) by one of the authors. Leaves were dried on silica for DNA extraction. Herbarium specimens are deposited in PRC, and duplicates stored in QCA, QCNE, and AAU. Further, we got additional material from AAU, B, MA, and BONN. We sampled 15 out of the 19 accepted *Loricaria* species and, in addition, four new morphological groups representing potentially new species (nine individuals; Supplementary Table 1).

For Hyb-Seq, we included between 1 and 13 samples per species to test for their monophyly, with a stronger focus on sect. *Thyopsis*, as it includes more widespread species than the other sections. The outgroup was complemented with sequences from Mandel et al. (2019), who used the same probe set for target enrichment (see below). In total, 13 species from seven genera of the Gnaphalieae were sampled as outgroup taxa. Overall, we sampled 63 individuals for this study, including 13 outgroup samples.

DNA extraction, genomic library preparation, and bait hybridization followed Gizaw et al. (2021). We used the Compositae1061 probe set (Mandel et al., 2014), implemented in the myBaits Expert Compositae1061 target capture kit (Arbor Biosciences, Ann Arbor, MI, United States). Enriched libraries were mixed with unenriched libraries in the ratio 2: 1 (run2), 1.5: 1 (run3), and 1: 1 (run5), respectively. Samples were sequenced on different sequencers, either an Illumina (San Diego, CA, United States) NextSeq at the Genomics Core Facility of CEITEC (Brno, Czechia) or an Illumina NovaSeq at IAB (Olomouc, Czechia); in all cases 150 base pairs (bp) paired-end reads were obtained. Raw reads are available under NCBI SRA BioProject PRJNA777419.

### Data Analysis Workflow

**FIGURE 1:**
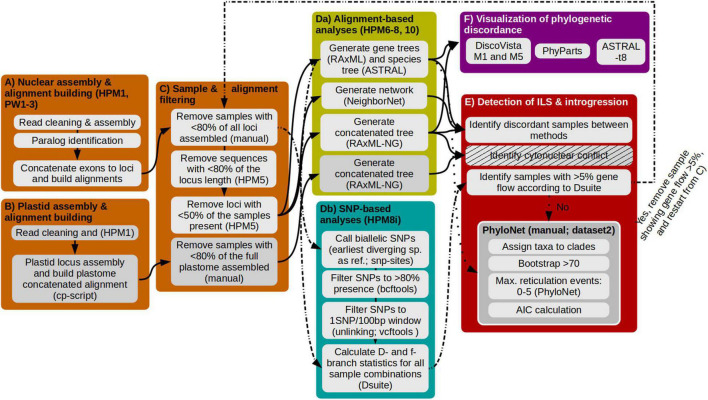
Illustration of the different steps employed for the discovery of samples and clades which show introgression to other clades. Each colored box represents a major analysis step, enumerated from **(A–E)**, analysis scripts used are indicated in the respective boxes in parentheses. **(A)** Assembly of nuclear reads, identification of paralogs, and alignment building. **(B)** Assembly of plastid reads and alignment building. **(C)** Filtration of alignments to exclude samples with few assembled exons and sequences that are too short. **(Da)** Calculation of gene and species trees. **(Db)** Identification and filtration of single nucleotide polymorphisms and analysis of gene flow. **(E)** Identification of ILS and introgression. At first, the pipeline follows the solid arrows which results in dataset 1. This dataset 1 is used to follow the dashed-dotted lines in an iterative approach to remove samples that show gene flow, finally resulting in dataset 2. Using dataset 2 and following the dotted line allows to identify if all hybridigenous samples have been detected. **(F)** Visualization of phylogenetic discordance between phylogenies is done for dataset 1 and dataset 2, respectively.

We developed a data analysis workflow that implements data filtering, paralog detection and utilization for phylogenetic reconstruction, and investigation of ILS and hybridization to robustly infer the phylogeny of a young radiating group (Figure 1). Most scripts used herein are part of HybPhyloMaker (Fér and Schmickl, 2018; scripts are available at https://github.com/tomas-fer/HybPhyloMaker), which we indicate as ‘‘HPM’’ followed by the number of the respective script. Customizing the reference, paralog detection, and orthologous alignment building was performed using ParalogWizard^1^ (scripts are available at https://github.com/rufimov/ParalogWizard), to which we refer to as “PW” followed by the number of the respective script. In the case of running scripts outside of these two bioinformatic pipelines we refer to the scripts directly in the respective methodological section, and if steps need to be done by the user manually, we denote this as “manual.” All steps and scripts are also summarized in the Supplementary Table 3.

#### Nuclear Read Assembly, Paralog Identification and Locus Alignment

The first part of the workflow will assemble nuclear reads, identify paralogs and align loci (Figure 1A). Raw reads were trimmed to remove adapters and low quality bases using Trimmomatic v.0.39 (Bolger et al., 2014) and duplicates were removed using BBMap v.38.42 (Bushnell, 2014) following the settings implemented in HPM 1: Low quality bases were considered to have a base quality encoding below 33 (phred33) and were coded as N. Additionally, we removed low quality bases at the beginning and end of the read if below Q20, and if bases in a sliding window of 5 bp were below the threshold Q20, the read was cut and the bases removed. Finally, we deleted reads shorter than 36 bp. Reads were assembled into contigs *de novo* using Compositae1061 exons as target file for initial read fishing during the assembly step (distribute_reads_to_targets_bwa.py and spades_runner.py from HybPiper v.1.3.1 [Johnson et al., 2016] implemented in PW 1a and b [see text footnote 1]). The minimum coverage to call a single nucleotide polymorphism (SNP) was set to 2. Subsequently, we customized the target file with sequences from our *Loricaria* reads (PW 2b) and repeated the mapping with this “Loricaria-optimized Compositae1061” target file (PW 1a and b), consisting of the best matching, longest exonic sequences from different *Loricaria* samples (see text footnote 1). Outgroup taxa were specified in the “blocklist” to exclude them in order to generate a target file containing only *Loricaria* sequences.

We followed (see text footnote 1; option 4 from the introduction) to detect paralogous loci and use both paralogs and orthologs for phylogenetic reconstruction. The approach is summarized in Gizaw et al. (2021). In brief, to assess paralogy, pairwise sequence divergence between the exonic contigs of each locus was estimated, which resulted in two main clusters of divergence, the first denoting allelic variation and the second paralogy. Similar to Gizaw et al. (2021), we chose the mean of the second cluster ± the standard deviation as the divergence threshold for considering exonic copies to be paralogous, using PW 2a. For our dataset, sequence divergence between 7.96 and 19.43% are considered to represent paralogous copies. Exonic alignments for each orthologous and paralogous copy were built using MAFFT v.7.029 (Katoh et al., 2005) and the exons concatenated to loci using PW 3.

#### Plastome Read Assembly

The second part of the workflow assembles the plastome and builds a concatenated alignment (Figure 1B). Plastome sequence data were obtained as a by-catch as the result of our adapted lab protocol that adds a proportion of unenriched libraries to the enriched libraries. Reads were trimmed for quality using Trimmomatic v.0.39 (Bolger et al., 2014) and duplicates removed using BBMap v.38.42 (Bushnell, 2014) as implemented in HPM 1. Detailed settings about read filtering are provided in the paragraph before. The remaining reads were mapped to a user-provided reference (*Leontopodium*; GenBank accession number NC027835) using BWA v.0.7.15 (Li and Durbin, 2009), implemented in a script available at https://github.com/tomas-fer/scripts/blob/master/cpDNA_mappingMETA.sh. Before the read mapping, one of the two inverted repeats of the plastome reference was removed using Geneious v.2020.1.2^2^. We then called the consensus sequence using kindel v.0.1.4 (Constantinides and Robertson, 2017) with a minimum read depth of 2 and with a 0.51 threshold for consensus variant calling; regions of the plastome without mapped reads were coded as N. The alignment was built using MAFFT.

#### Sample and Alignment Filtering

The third part of the worklow filters samples and alignments with too much missing data (Figure 1C). As missing data have a substantial impact on correct species tree estimation, especially under a high degree of ILS (Nute et al., 2018), we tested different subsampling strategies toward optimally incorporating poorly assembled samples into the nuclear and plastome phylogenies, respectively (hereafter referred to as low quality samples): not excluding them, excluding samples with less than 50% assembled loci, and with less than 80% assembled loci. Based on the number of loci recovered per sample that is reported in the table ‘MissingDataOverview.txt’ (created by HPM 5), we deleted samples manually from the analyses folder before continuing. We aimed at an optimal tradeoff between the number of assembled loci per sample and the number of samples removed from the dataset due to low quality. As such, the nuclear and plastome datasets include slightly different sets of samples.

We employed a second filtering step, this time for the nuclear alignments only. For each locus, we excluded sequences missing more than 50% data for the locus, and we removed loci for which less than 80% of all samples were represented (HPM 5).

#### Alignment- and Single Nucleotide Polymorphism- Based Analyses and Identification of Incomplete Lineage Sorting and Introgression

The fourth part of the workflow consists of alignment- and SNP-based analyses (Figure 1D). Based on the filtered alignments from the step above (hereafter referred to as dataset 1), we inferred phylogenetic hypotheses using two methods, the MSC method (nuclear dataset only) and concatenation (Figure 1Da). For the MSC method, gene trees were estimated using RAxML v.8.4.2 (Stamatakis, 2014) with the general-time reversible (GTR) substitution model with a gamma distributed rate variation among sites “GTRGAMMA” and 500 bootstrap replicates (HPM 6a). Based on these gene trees, we generated an ASTRAL species tree using ASTRAL III v.5.6.1 (Zhang et al., 2017; HPM 8a). For the species tree calculation, we initially tested the effect of collapsing poorly supported gene tree nodes (HPM 10). This showed no effect in our dataset, but we recommend to test that during analysis. Additionally, all loci were concatenated into a locus-partitioned supermatrix (HPM 8f) and a phylogeny was inferred using RAxML-NG v.8 (Kozlov et al., 2019; run manual), hereafter referred to as concatenated tree. For the plastome, the same concatenation approach, but without partitioning was utilized.

In a next step, the datasets were evaluated for signatures of ILS and hybridization (Figure 1E). The first round of evaluation employed commonly used approaches, which do not provide full evidence of ILS and/or hybridization. Incongruent placement of samples between the following phylogenetic comparisons are commonly treated as an indication of ILS and/or hybridization: First, based on the nuclear phylogenetic reconstructions we identified incongruent placements between the ASTRAL species tree and the concatenated tree. Second, based on a comparison between the plastome and the nuclear (ASTRAL) phylogeny we inferred cytonuclear discordance. In addition, a distance-based network was generated using NeighborNet (Bryant and Moulton, 2004) available in SplitsTree v.4.16.2 (Huson, 1998; HPM 8 h). Admixed samples were determined to be those forming mixed groups (i.e., samples grouping with different samples in the NeighborNet compared to well-supported clades in the phylogenetic results) or showing a misplacement in the network (i.e., isolated samples).

The second round of evaluation was based on full-evidence approaches that simultaneously account for ILS and introgression: Dsuite (HPM 8i; Figure 1Db) and PhyloNet (run manual; Figure 1E). Using Dsuite v.0.4r38 (Patterson et al., 2012; Malinsky et al., 2021; HPM 8i), we calculated the D-statistics (also called ABBA-BABA statistics) for all trios (with a fixed outgroup) of species in the dataset and f-branch statistic to evaluate the amount of introgression. The D-statistics estimates the frequency of “ABBA” and “BABA” patterns in a four-taxon phylogeny {[(Sample1,Sample2)Sample3]Outgroup}, whereas the SNP “A” denotes the ancestral SNP and “B” the derived. Under ILS, both patterns are equally likely, whereas an excess of one pattern indicates introgression. To perform the analyses, we called SNPs for the ingroup based on *L. graveolens* (sample LR_017; sister to the remaining species in the phylogeny) as reference using snp-sites v.2.3.3 (Page et al., 2016). Before SNP calling we concatenated all loci irrespective of missing data. We retained only biallelic SNPs, and removed SNPs with more than 20% missing data using bcftools v.1.7 (Li, 2011), leading overall to more SNPs than if we would have used the alignments after the missing data filtering step. To only retain unlinked SNPs, we then filtered the biallelic SNPs for one SNP per 100 bp window using vcftools v.0.1.17 (Danecek et al., 2011), resulting in about 1500 SNPs in total from initial 16,000 SNPs. To run the Dsuite analysis, the ASTRAL species tree was used as input. Dsuite allows to assign samples to species to account for intra-specific variation, but we decided to map samples to samples, as most species were not retrieved as monophyletic, and we did not know if this was due to a hybridigenous origin of these species or of certain samples. We will use the term ‘hybridigenous origin’ throughout the manuscript in cases where we are not able to distinguish between introgression and hybridization. Samples that showed more than 5% introgression to samples from different clades based on the f-branch statistic (introgression cut-off according to Malinsky et al., 2021) were removed and the species tree recalculated. We employed an iterative approach by rerunning the analysis until no major events of introgression (>5%) could be detected, as we found that Dsuite continued detecting samples with signatures of introgression to other samples after removing the first set of samples showing introgression. Removal of all introgressed samples resulted in a reduced dataset (hereafter referred to as dataset 2).

To test if the Dsuite analyses detected all introgressed samples and to reveal if the evolutionary structure of *Loricaria* is tree-like, we applied evolutionary network analyses implemented in PhyloNet v.3.8.2 (Than et al., 2008) using the computationally least demanding maximum pseudo-likelihood (MPL) approach. PhyloNet returns the degree of gene flow, denoted as γ-parameter, of the two parents to the hybrid. We used all gene trees after removing samples that showed introgression according to the Dsuite analyses (dataset 2), and only considered nodes with a bootstrap support greater than 70% (nodes were collapsed prior to analysis using HPM 10). We allowed between zero to five hybridization events. Samples were mapped to supported clades; without this data simplification, none of the analyses finished within 2 weeks calculation time employing 14 CPUs. We then performed a model selection using the Akaike information criterion (AIC); the best supported network is the one with the lowest AIC value. The number of parameters for the AIC calculation equals the number of branches plus the number of allowed reticulation events and number of gene trees used to estimate the likelihood.

#### Visualization of Gene Tree Discordance Across Different Datasets

As the sixth part of the workflow, gene tree discordance is measured between phylogenies using different approaches (Figure 1F). First, we used PhyParts based on all gene trees and a node-support threshold of 70% (Smith et al., 2015; HPM 11). PhyParts calculates the number of concordant as well as discordant nodes between gene trees in comparison to the species tree. Second, we used the quartet-based method provided in ASTRAL III (the -t 8 option; run manual) to identify the percentage of alternative quartets. For the quartet-based method, an equal proportion of quartets indicates a high degree of ILS (Sayyari and Mirarab, 2018). Third, in contrast to the above mentioned methods, to differentiate between highly and moderately conflicting nodes that conflict between gene trees and the species tree and, hence, to provide a more detailed understanding of the gene tree discordance we used DiscoVista (Sayyari et al., 2018; run manual). DiscoVista also permits to visualize differences in gene tree discordance between our different sample and alignment filtering approaches. The discordance analysis on gene trees (method 1 [M1] in Figure 1F) and the relative frequency analysis of alternative topologies (method 5 [M5] in Figure 1F) were based on a support threshold value of 70% and a maximum of 20% missing samples in the clade.

## Results

### Assembly, Alignment, and Alignment Filtering

As missing data have a substantial impact on correct species tree estimation, especially under a high degree of ILS (Nute et al., 2018), we chose a relatively stringent tradeoff between the number of assembled loci per sample and the number of samples removed from the dataset due to low quality. We excluded samples with a recovery of less than 80% across all loci from both the nuclear and plastome datasets, which resulted in the removal of 26 and 28 samples for the nuclear and plastome dataset, respectively. In total, we used three outgroup samples and 34 samples from the ingroup for the nuclear dataset, reducing the dataset from 63 to 37 samples. For the plastome dataset, we used 10 outgroup samples and 25 samples from the ingroup, totaling 35 samples. Alignments were built for each dataset separately, and after sample filtering resulted in 13 species plus our additional four new morphological groups for the nuclear alignments, and 13 species plus additionally three of the new morphological groups of *Loricaria* for the plastome alignments.

After trimming and de-duplicating the raw reads, between 0.9 and 14 million reads per sample were retained. The nuclear dataset 1, from which the initial ASTRAL species tree was reconstructed, consisted of 973–1150 loci per sample. To avoid data loss by removing paralogous loci, we differentiated between orthologous and paralogous copies and built separate alignments from those, which increased the number of loci for phylogenetic reconstruction by about 25%. We did not find a higher degree of paralogy in particular species or clades (Supplementary Table 2).

**FIGURE 2:**
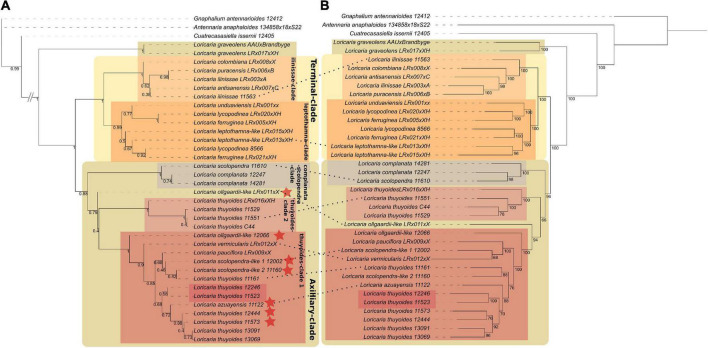
Nuclear species tree using ASTRAL III **(A)** in comparison to the concatenated tree calculated using RAxML-NG (**B**; dataset 1). Values at nodes in **(A)** represent local posterior probabilities and in **(B)** bootstrap values. Samples that differ in their phylogenetic placement are indicated by a dotted line. Red stars indicate samples that got removed from dataset 2. Labeled boxes indicate major clades discussed in the main text. Red unlabeled boxes indicate *L. thuyoides* samples with a different placement in the plastome tree (see Supplementary Figure 4).

Without employing our stringent sample filtering by removing samples with less than 80% of the exons recovered, the support for clades within *Loricaria* was low [<0.95 local posterior probability (LPP); data not shown]. After removing those samples (HPM 5), the number of loci used during alignment building increased, as these low-quality samples did not longer have a dominating effect on the removal of loci that had less than 80% of all samples present (Supplementary Figure 1A). Further, gene tree discordance decreased (Supplementary Figure 1A), and the support for the major clades (those discussed later) increased, from only four nodes within *Loricaria* having a support greater than 0.95 LPP to eight such nodes (Figure 2A; not counting supported nodes within species-complexes).

The number of mapped plastid reads ranged from 5027 to 199,001, with an average proportion of missing data of 3.3% (min-max: 0.06–17.6%). The length of the concatenated plastome dataset after removing samples with more than 20% missing data was 261,701 bp.

### Phylogenetic Analyses and Testing for Signatures of Incomplete Lineage Sorting

The monophyly of *Loricaria* was strongly supported based on the nuclear data, both in the ASTRAL species tree (1 LPP) and the concatenated tree (100% bootstrap support [BS]; Figure 2; dataset 1). The earliest diverging taxon to the outgroup, *L. graveolens*, the only representative of the sect. *Graveoleum*, was the only species with support for monophyly (ASTRAL: 1 LPP; concatenation: 100% BS). Based on the ASTRAL and concatenated tree the two main clades retrieved grouped species of high morphological similarity (for dataset 1: Figure 2; for dataset 2: Supplementary Figure 6), and we hereafter refer to these main clades as the Terminal- (1 LPP; 100% BS for dataset 1 and 2) and Axilliary-clade (0.88 LPP/0.96 LPP for dataset 1/dataset 2 and 96%/76% BS, respectively). The phylogenetic placement of most samples followed the sectional classification of the genus, with the exception of *L. unduaviensis* Cuatrec. and one new morphological group, *L. “leptothamna-like,”* which were placed with samples from the sect. *Terminalia*. *Loricaria unduaviensis* and *L. leptothamna* were placed in the sect. *Thyopsis* by Cuatrecasas (1954), irrespective of the position of their capitulum, which is terminal, not axilliary. Similarly, *Loricaria colombiana* has axilliary capitulas, but is part of the Terminal-clade and assigned to sect. *Terminalia*. The samples identified as *L. “ollgaardii-like,”* have high morphological similarity to *L. ollgaardii* except for the position of their capitulas, which is axilliary in our samples but terminal in the original species description. Accordingly, our samples were found in the Axilliary-clade. In addition to the *L. “ollgaardii-like”* samples, the Axilliary-clade consisted of two subclades, the scolopendra-complanata-clade (1 LPP; 100% BS for dataset 1 and 2) and the thuyoides-complex, which comprised samples from *L. pauciflora* Cuatrec., *L. azuayensis* Cuatrec., and *L. thuyoides* as well as three of the four new morphological groups. Within the thuyoides-complex, samples of the species *L. thuyoides* were found to be non-monophyletic: samples of *L. pauciflora* and *L. azuayensis* were nested among *L. thuyoides* samples. Further, four *L. thuyoides* samples formed a sister clade relationship to the remaining samples of the Axilliary-clade (1 LPP; 100% BS for dataset 1 and 2), denoted as thuyoides-clade2. The Terminal-clade comprised the ilinissae-clade (1 LPP; 100% BS for dataset 1 and 2), including *L. ilinissae* (Benth.) Cuatrec., *L. puracensis Cuatrec., L. antisanensis* Cuatrec., and *L. colombiana* Cuatrec., and the leptothamna-clade (dataset 1: 0.99 LPP; 100% BS; dataset 2: 0.97 LPP; 100% BS), composed of *L. “leptothamna-like,” L. lycopodinae* Cuatrec., *L. ferruginea* (Ruiz & Pav.) Wedd., and *L. unduaviensis.*

**FIGURE 3:**
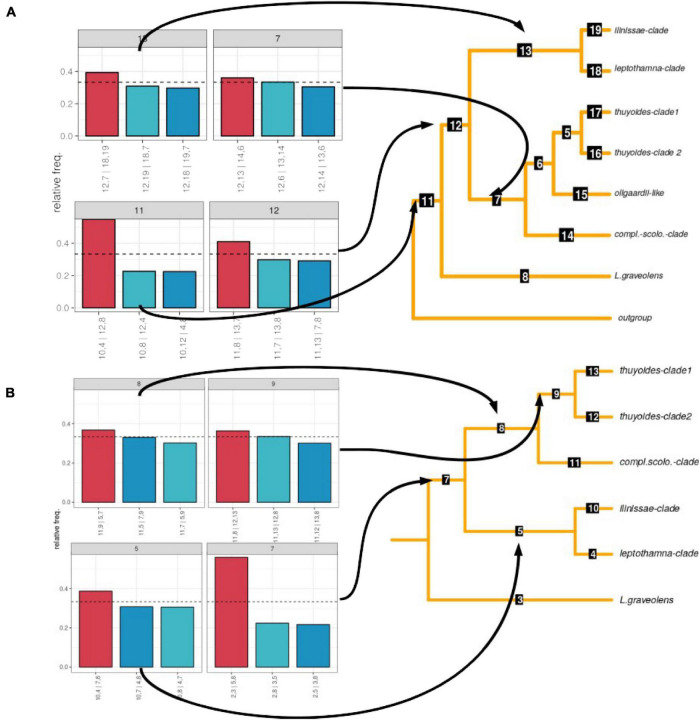
Frequency of alternative topologies supported by gene trees before (dataset 1; **A**) and after removing samples that showed gene flow according to Dsuite (dataset 2; **B**). The relative frequencies of the topologies are shown on the left. On the right, the main topologies are shown that are reduced to clades; the numbers on the branches indicate node numbers.

We recovered the same clades for the concatenated phylogeny as we did based on the ASTRAL species tree, with only a few samples showing different placements within these clades (Figure 2). Analysis of gene tree discordance using PhyParts showed that each node is highly discordant, with only a small proportion of gene trees supporting the species tree (Supplementary Figure 2). When investigating the frequency of the different topologies retrieved among the gene trees using DiscoVista (method 5), for the node leading to the genus, one topology was most frequent (>50%, Figure 3A). After removing samples that showed gene flow (dataset 2), the split separating the early diverging species *L. graveolens* from the remaining clades was found in more than 50% of the topologies (Figure 3B). The remaining nodes showed almost equal frequencies for all three topologies (highest frequency of maximally 40%; Figure 3A). The percentage of alternative quartets according to ASTRAL gave similar results, mostly having similar frequencies for all three quartets (Supplementary Figure 3). Only the node indicating genus monophyly and the species *L. graveolens* were well supported with more than 50% of the quartets showing the same topology. Clades supported by more than 40% of the quartets were the ilinissae-clade, the scolopendra-complatana-clade, and the thuyoides-clade2, as well as the mrca of the Axilliary- and Terminal-clade (Supplementary Figure 3).

### Testing for Signatures of Introgression

Cytonuclear discordance was strong in our dataset (Supplementary Figure 4). The plastome tree was reconstructed using a slightly different selection of samples than for the nuclear trees, due to our missing data filter approach. While most clades recovered in the nuclear dataset were present in the plastome tree, there were major differences. First, the genus was non-monophyletic: *Belloa schultzii* (Wedd.) Cabrera formed a clade with the earliest diverging species *L. graveolens* (100% BS). Second, the only sample available from the scolopendra-complanata-clade (14281) for the plastome dataset plus two samples of *L. thuyoides* (11523, 12246) formed a clade together with the samples from the ilinissae-clade (100% BS), while other members of the thuyoides-clade1 did not (Supplementary Figure 4). Additionally, the ilinissae-clade is part of the Axilliary-clade in the plastome tree (100% BS).

According to the distance-based network, we identified a different set of misplaced samples compared to the ones identified in the plastome tree as well as those that showed signs of ILS: Samples from two of the new morphological groups, i.e., *L. “ollgaardii-like”* samples (12066, LR_011) and the *L. “scolopendra-like 1”* sample (12002; Supplementary Figure 5) grouped with different samples in the network than in the phylogenetic reconstructions.

Calling SNPs from our Hyb-Seq data resulted in an initial set of 16,000 SNPs before filtering according to our minimum threshold for SNP presence across samples and accounting for linkage. Approximately 50% of the SNPs were removed due to their presence in less than 80% of the samples, and restricting the SNP dataset to unlinked SNPs resulted in a further reduction to final 1515 SNPs. Based on the Dsuite analyses, we identified seven samples showing introgression greater than 5% (Table 2). All these samples belonged to the thuyoides-clade1: three out of the four new morphological groups (*L. “ollgaardii-like”*: 12066, LR_011; *L. “scolopendra-like 1”*: 12002; *L. “scolopendra-like 2”*: 11160), two samples of *L. thuyoides* (12444, 11573), and *L. azuayensis* (11122).

**TABLE 2:** Summary of all methods that were used to detect samples showing phylogenetic discordance.

Clade	Discordant taxon	ILS	Cytonuclear discordance	NeighborNet	Introgressed clades (PhyloNet)	Degree of introgression (Dsuite sub-analysis)
Ilinissae-clade	*L. ilinissae* 11563	x				
			
	*L. antisanensis* LR_007		x			

Leptothamna-clade	*L. leptothamna-like* LR_013_XH	x				
			
	*L. lycopodinae* LR_020		x			

Complanata-scolopendra-clade	*L. scolopendra* 11610	x	–		o	
			
	*L. complanata* 14281		o			

	*L. ollgaardii-like* LR_011_X	x	x	o		0.07 (2)

	*L. ollgaardii-like* 12066		–	o		0.045 (5)

Thuyoides-clade1	*L. vermicularis* LR_012_X	x	–			
			
	*L. azuayensis* 11122	x	–			0.06 (5)
			
	*L. scolopendra-like 1* 12002	x	–	x		0.12 (3)
			
	*L. scolopendra-like 2* 11160		x			0.14 (1)
			
	*L. thuyoides* 11161	x			o	
			
	*L. thuyoides* 12444		–			0.10 (4)
			
	*L. thuyoides* 11551	x				
			
	*L. thuyoides* 11573					0.06 (2)
			
	*L. thuyoides* 12246		o			
			
	*L. thuyoides* 11523		o			

Thuyoides-clade2					o	

*A dash (“–”) indicates that the sample is absent from both phylogenies used for the comparison. The letter “o” indicates the placement in a different clade, the letter “x” indicates a different position within a clade. The degree of introgression using the f–branch statistic in Dsuite is only presented when values were γ > 0.05. In such cases, the number of the sub-analysis in which gene flow was detected is indicated in parentheses.*

While the samples identified as discordant varied between the different methods, they all belonged to the Axilliary-clade (Table 2). The Dsuite analyses indicated that most samples showed introgression with members of the ilinissae-clade. Removing those samples (dataset 2) reduced gene tree discordance for the mrca of the Terminal- and Axilliary-clade (Figure 3B) and increased support for the phylogenetic backbone (Supplementary Figure 6).

**FIGURE 4:**
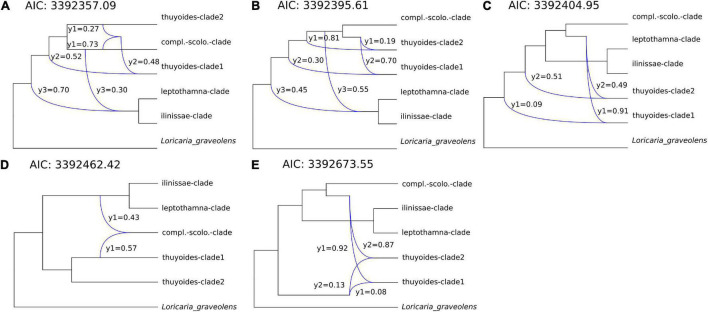
(**A–E**) show the five best inferred networks with a maximum of three reticulation events according to PhyloNet using all gene trees (dataset 2). Phylogenies were reduced to clades and only nodes above a bootstrap value of 70% were considered. Gene flow between lineages is indicated by a blue line leading from two lineages to a third one. The γ-parameter indicates the degree of gene flow between different lineages. AIC values are presented and also provided in Supplementary Table 4.

Only after removing those samples showing introgression with samples from different clades (dataset 2) were the PhyloNet analyses able to finish. Thus, we used PhyloNet to test if all potential hybridization events were detected using Dsuite. Allowing for a maximum of three hybridization events resulted in the best model according to the AIC in PhyloNet (Supplementary Table 4). The structure of the five best networks within this analysis was mostly consistent, indicating that within *Loricaria* there were between one to three reticulation events, the γ-parameter ranging from 0.9 to 0.49, resulting in a hybridigenous origin of either the thuyoides-clade1 or -clade2 and/or the complanata-scolopendra-clade with an ancestor of the Terminal-clade (Figure 4). In four out of the five best networks, six out of 11 introgression events showed γ ≥ 0.3 within at least one event per network (Figure 4); the remaining events showed lower levels of gene flow.

## Discussion

### Utility of Universal Probe Sets for Resolving Recent Rapid Radiations

Target enrichment protocols employing probe sets that may be either customized (often genus- or tribe-specific; designed to match exons across a relatively small number of species) or universal (family- or order-specific; designed to match exons across larger ranges of the tree of life) are widely used to generate hundreds of nuclear loci for samples from evolutionary lineages ranging from deep to shallow phylogenetic scales (e.g., Carlsen et al., 2018; Johnson et al., 2019; Bagley et al., 2020). Although custom and universal probes often show a similar relative performance (Larridon et al., 2020; Shah et al., 2021; Ufimov et al., 2021), universal probes are frequently preferred for non-model organisms, for which the genomic resources, which are a prerequisite for the probe design, are often not available and too costly to generate.

In this study, we used the universal probe set Compositae1061 (Mandel et al., 2014), but customized the reference for “read fishing” before *de novo* assembly, which likely increases locus recovery and the length of recovered exons, as was shown in McLay et al. (2021) and Ufimov et al. (2021). (Universal) target enrichment kits target conserved exons that are present across a wide range of taxa. Hence, the phylogenetic signal is reduced, especially compared to introns (Folk et al., 2015; Bagley et al., 2020; Gardner et al., 2021). Nevertheless, the utility of the Compositae1061 probe set to resolve young phylogenies was shown before (Gizaw et al., 2021). By using ParalogWizard to detect paralogous loci and utilize them for phylogenetic reconstruction, we restricted our data analysis to exons, as ParalogWizard was developed for exons only. In contrast, HybPiper, the standard data analysis pipeline for Hyb-Seq data, permits to use flanking introns or supercontigs (exons plus flanking introns), potentially providing more phylogenetic informative characters (Jones et al., 2019; Ogutcen et al., 2021; Ufimov et al., 2021). However, an earlier study showed that for *Antennaria*, a close relative of *Loricaria*, very few supercontigs remained after alignment trimming and the remaining ones had low degrees of informative characters, a pattern generally prominent for Gnaphalieae (Jones et al., 2019).

While nuclear Hyb-Seq data are often able to resolve phylogenetic relationships with high support, gene tree discordance is usually high in these datasets (Jones et al., 2019; Smith et al., 2020), and the species tree topology can even be represented by only a minority of gene trees (so-called anomaly zone; Degnan and Rosenberg, 2009; Liu and Edwards, 2009; Roch and Steel, 2015). The degree of gene tree discordance tends to be lower for custom probe sets (Bagley et al., 2020; Siniscalchi et al., 2021), especially if the custom probes target longer loci compared to the universal probes (Ufimov et al., 2021). In the case of *Loricaria*, we found a high degree of gene tree discordance also in comparison to other young Asteraceae groups. However, we were able to show that using stringent filters for missing data and an elaborate analysis workflow can reduce gene tree discordance, and at it least partly explains its underlying biological processes.

Repeated rounds of WGD are common for the angiosperms (Wendel, 2015), also for the tribe Gnaphalieae and family Asteraceae (Smissen et al., 2011; Barker et al., 2016; Huang et al., 2016; Zhang et al., 2020b). We, thus, accounted for paralogy during alignment building to remove this source of gene tree discordance before addressing the effect of ILS and hybridization on discordance. Even though the Compositae1061 probe set was designed to comprise exclusively single-copy loci, a certain proportion of the loci were flagged as paralogous using HybPiper in recent works (Jones et al., 2019; Siniscalchi et al., 2019). We inferred that about 25% of the loci included paralogous copies in our dataset. The high number of paralogous loci can likely be attributed to the multiple WGD events within the family Asteraceae and the tribe Gnaphalieae in particular. As previous genome size estimates indicated that the genus lacks neopolyploids (Kolář et al., 2016), the duplicated loci are likely the result of WGDs in the tribe.

It should be noted that we do not address certain methodological artifacts as sources of gene tree discordance, namely the effect of (a) collapsing weakly supported nodes in gene trees (HPM 10), (b) removing gappy regions in the alignment (HPM 4a3), (c) selecting the most parsimony informative alignments (run manual) or (d) excluding loci showing signs of recombination (PhiPack; Bruen et al., 2006; run manual). Initial analyses showed that the effect of these artifacts on gene tree discordance was weak for our datasets (data not shown). While gene tree discordance tends to decrease with increasing data completeness (Siniscalchi et al., 2019), our stringent removal of low-quality samples and alignment filtering likely reduced the possible effect of methodological artifacts on gene tree discordance to a minimum (Supplementary Figure 1). Although several highly interesting samples were removed in the process of filtering for missing data, it was the only possibility to reduce gene tree estimation errors and, thus, be able to focus on the biological processes as sources of gene tree discordance (Supplementary Figure 1).

### Phylogenetic Relationships in *Loricaria*

The genus *Loricaria* is monophyletic according to our nuclear phylogeny (Figure 2), while *Belloa* is nested within the earliest diverging lineage of *Loricaria* according to the plastome phylogeny (Supplementary Figure 4). Employing a less stringent sample filter allowed to include *Belloa* in the nuclear dataset, and in this case *Belloa* did not belong to the genus *Loricaria* in both the ASTRAL species tree and concatenated tree (data not shown). The placement of *Belloa* within *Loricaria* based on the plastome dataset provides evidence for ILS or hybridization between lineages across genera in the Gnaphalieae. This highlights that a well-sampled outgroup and good knowledge about sister lineages through broader sampling of the tribe is required to gain a better understanding of the evolution of the genus and the tribe.

The phylogeny is split into three major clades, reflecting mainly the three different sections within the genus. Strong support for species monophyly was only found for *L. graveolens*, whereas all other species were not supported to be monophyletic. Whether this is due to gene flow between species, overdescription of taxonomic species or limited phylogenetic signal for young high-altitude Andean groups for the universal Compositae1061 loci needs to be evaluated. The widespread species *L. thuyoides* is highly polyphyletic, as indicated in Kolář et al. (2016). We detected introgression between seven members of the thuyoides-clade1 and members of the ilinissae-clade using Dsuite, and another three samples showed strong cytonuclear conflict (Supplementary Figure 4). These results were confirmed by the PhyloNet analyses. The high degree of gene tree discordance (Supplementary Figure 1), while accounting for paralogy due to WGD during alignment building, suggests that ILS and hybridization played an important role in the evolution of *Loricaria*.

### Signatures of Incomplete Lineage Sorting in *Loricaria*

Although the initial species tree after sample and alignment filtering (dataset 1) supported most of the major clades in the phylogeny with support > 0.95 LPP, three different gene tree topologies were almost equally likely for most nodes, indicating a substantial degree of ILS (Figure 2A). It needs to be noted that a comparison between the ASTRAL species tree and the concatenated tree resulted in the same set of supported clades, although within these clades some samples showed different placements (Figure 2). This low discordance indicates a moderate degree of ILS, as RAxML is more sensitive to ILS than ASTRAL (Mirarab et al., 2016). Collapsing nodes with low support (i.e., 10 or 30% BS) before species tree calculation did not decrease the degree of discordance (data not shown), indicating that the discordance is not due to low phylogenetic signal, but rather due to ILS.

In rapidly radiating lineages, the degree of ILS is expected to be high (Whitfield and Lockhart, 2007), due to insufficient time for alleles to coalesce. Earlier molecular dating efforts estimated a crown age of 4 Ma for *Loricaria* (Nie et al., 2016), which resulted in an approximate net diversification rate of 0.74 species per Million years [ln(N)/t; N: number of species, t: crown age; Magallon and Sanderson, 2001]. This is comparable to the high diversification rates for other plant groups from tropical high elevations in South America (Madriñán et al., 2013), as well as rates that were found in the biodiversity hotspot of the Cape Floristic Region (Pirie et al., 2016).

The degree of ILS increases with large population sizes (Slatkin and Pollack, 2006). *Loricaria* evolved during the uplift of the Andes in the northern parts of South America, where during Pleistocene glaciations the alpine belt shifted downwards (Van der Hammen, 1985; Hooghiemstra and Van der Hammen, 2004), resulting in larger potentially suitable habitats. This might have resulted in larger effective population sizes during these intervals throughout the evolution of *Loricaria.* In a future study, we will investigate the demography of *Loricaria* species using a population genomics approach.

### Signatures of Hybridization in *Loricaria*

The sum of our results (cytonuclear discordance, the NeighborNet as well as the Dsuite and PhyloNet analyses) indicate that ILS alone cannot explain the high degree of discordance that we observed within *Loricaria* (Figure 2, Table 2, and Supplementary Figure 2). The removal of samples with introgression according to Dsuite (dataset 2) increased the support for the major split between the two main subclades in the genus from about 40% of all gene trees to above 50% (Figure 3 and Supplementary Figure 2). Nevertheless, standard methods to illustrate genomic discordance did not show major improvements (based on node support and PhyParts; Figure 2 and Supplementary Figures 1B, 2) between dataset 1 and dataset 2. This highlights the importance to investigate species tree hypotheses beyond support values and standard methods of measuring discordance and to thoroughly test for all potential sources of discordance, especially in highly understudied lineages.

Hybrids are unknown for the genus *Loricaria* based on morphological evidence (Cuatrecasas, 1954). In addition, previous genome size estimations of several *Loricaria* species revealed only relatively small differences (8.76–11.69 pg DNA; measurements available for *L. ilinissae, L. scolopendra, L. thuyoides*, and *L. complanata*; Kolář et al., 2016), suggesting the absence of hybridization, under the assumption that hybrids are frequently stabilized by polyploidization. Using a diverse spectrum of methods, we detected multiple hybridization events within *Loricaria.* This was not surprising given that the genus belongs to the tribe Gnaphalieae, for which many hybridization events have been reported (e.g., Smissen et al., 2011; Galbany-Casals et al., 2014; Barker et al., 2016; Huang et al., 2016; Vargas et al., 2017; Watson et al., 2020; Zhang et al., 2020b). Further, several radiating plant groups in the tropical high altitude areas of South America show hybridization (*Lachemilla*: Morales-Briones et al., 2018; *Lupinus*: Nevado et al., 2018; *Diplostephium*: Vargas et al., 2017; Espeletiinae: Cortés et al., 2018). The dynamic nature of this ecosystem with multiple range expansions and contractions during the Pleistocene (Flantua et al., 2019) may have facilitated the contact between geographically isolated species that probably did not yet exhibit strong barriers to gene flow. Using Dsuite, we identified a total of seven samples showing introgression with samples of other clades in the species tree. After removing those samples, we were able to detect one to three clades within the genus that are of hybridigenous origin according to PhyloNet (Figure 4). Unfortunately, PhyloNet and related methods (SNAQ; Solís-Lemus et al., 2017) are difficult to use for large datasets with hundreds of samples and a high number of hybridization events. We ran PhyloNet using the MPL algorithm, after unsuccessfully attempting to utilize the “divide and conquer” method (Zhu et al., 2019) using a maximum likelihood implementation, which did not finish (<14 days and 14 CPUs of computation) for a subset of quartets, even though the method is intended for large datasets. The subset of quartets that did not finish included to a large extent those that were subject to hybridization events based on MPL (observations during trials). While PhyloNet accounts for gene flow and ILS as a source of discordance, the type of gene flow can be the result of hybridization, introgression or horizontal gene transfer. These processes are biologically very similar and cannot be differentiated methodologically by this method. However, different degrees of gene flow between species or lineages, depicted by the γ-parameter, may hind-cast the different processes (Solís-Lemus et al., 2017), in our case supporting hybridization between early lineages within *Loricaria* (six out of 11 introgression events showed γ ≥ 0.3, Figure 4). Nevertheless, the different degree of gene flow detected, ranging from γ = 0.09–0.49, suggests pure hybrids as well as hybridization with extensive backcrossing.

The polyphyletic nature of *L. thuyoides*, with some samples showing cytonuclear discordance, and others exhibiting introgression with members of the ilinissae-clade according to Dsuite analyses, indicates that *L. thuyoides* was subject to chloroplast capture and hybridization early in its history. While *L. thuyoides* is described to be morphologically variable (Cuatrecasas, 1954), we could not find any morphological characters that enable samples showing introgression to be distinguished from pure samples. Chloroplast capture is the result of two species hybridizing with extensive backcrossing to one of the parents (Rieseberg and Soltis, 1991). Due to the extensive backcrossing, the nuclear signal of the hybridization event is swamped out, but the novel plastid from the hybridization event remains. The two *L. thuyoides* samples showing indication of chloroplast capture group in the plastome phylogeny with ilinissae-clade samples from close geographic proximity, a pattern common for chloroplast capture (Acosta and Premoli, 2010; Liu et al., 2020). The clades we identified to have a potentially hybridigenous origin, the scolopendra-complanata-clade and both thuyoides-clades (Figure 4), overlap geographically with members of the ilinissae-clade, the potential hybridization partner.

The samples of the Axilliary-clade, which showed signatures of introgression according to the Dsuite, are predominantly found in southern Ecuador, close to the Huancabamba Depression in northern Peru, which exhibits a partial interruption of the Andes by low-elevation river systems. Some works suggested that this area poses a barrier to gene flow for high altitude species (Cosacov et al., 2009; Richter et al., 2009), a pattern that cannot be confirmed by our study. Members of the two main clades, the Terminal- and Axilliary-clade, are found on both sides of this depression. Further, two of the new morphological groups were found in close proximity to this area, *L. “vermicularis” and L. “ollgaardii-like,”* and the latter was found to be of hybridigenous origin. Due to the shifts of the alpine belt during the Pleistocene glaciation and deglaciation cycles (Flantua et al., 2019), populations of the different clades likely came into contact in the Huancabamba Depression, which might have facilitated hybridization (L. *“ollgaardii-like,” L. “scolopendra-like” 1 and 2*) as well as speciation (*L. “vermicularis”)*. As such, for species of *Loricaria* that are exclusively found in the páramo ecosystem, the Huancabamba area does not seem to be a barrier to gene flow. The Huancabamba Depression has also been identified as a center of diversity for montane species (Mutke et al., 2014; Quintana et al., 2017). We cannot confirm, however, if this secondary contact was also facilitated in the southern part of the Huancabamba Depression, as we lack good sampling from the northern parts of Peru.

Despite evidence for hybridization within *Loricaria*, the exact parents and clades subject to the hybridization events could not be determined. Due to our simplification in the PhyloNet analysis, by mapping samples to supported clades, we could not differentiate if only some of the species in the clade or the clades as a whole were subject to the hybridization events. To elucidate which species are of hybrid origin and which parental species gave rise to these hybrids, further sampling in the area is needed as well as population-level analyses.

Hundreds of loci and thorough testing of potential causes of discordance provided a better understanding of the evolution of the genus. And yet, while nowadays detecting hybrids using genomic data is easier than during Sanger sequencing times, the lack of knowledge about lineages and missing taxonomic expertise in young radiations complicate our understanding of their evolution.

## Data Availability Statement

The data generated for this study can be found in Genbank SRA under Bioproject number: PRJNA777419 (https://www.ncbi.nlm.nih.gov/bioproject/?term=PRJNA777419).

## Author Contributions

RS, MK, PS, and FK conceived and designed the research. PS performed the fieldwork and curated the plant material. MK processed the data, performed the phylogenetic analyses, and led the manuscript preparation. RS supervised the analyses and improved the manuscript. RS and FK facilitated the project by logistic and infrastructure support. All authors contributed to the article and approved the submitted version.

## Conflict of Interest

The authors declare that the research was conducted in the absence of any commercial or financial relationships that could be construed as a potential conflict of interest.

## Publisher’s Note

All claims expressed in this article are solely those of the authors and do not necessarily represent those of their affiliated organizations, or those of the publisher, the editors and the reviewers. Any product that may be evaluated in this article, or claim that may be made by its manufacturer, is not guaranteed or endorsed by the publisher.

## Footnotes

1 Ufimov, R., Gorospe, J. M., Fér, T., Kandziora, M., Salomon, L., van Loo, M., et al. (in prep). Utilizing paralogs for phylogenetic reconstruction has the potential to increase support and reduce gene tree discordance in target enrichment data. In preparation for Molecular Ecology Resources.

2
www.geneious.com
