# Supplementary material for: How to Tackle Phylogenetic Discordance in Recent and Rapidly Radiating Groups? Developing a Workflow Using Loricaria (Asteraceae) as an Example
Source: Front Plant Sci. 2022 Jan 7;12:765719. doi: 10.3389/fpls.2021.765719 (PMC8777076; doi:10.3389/fpls.2021.765719)
Supplement: Supplementary file 1 [file Data_Sheet_1.pdf]

Supplementary Material to
How to tackle phylogenetic discordance in recent and rapidly radiating groups? Developing a workflow using *Loricaria* (Asteraceae) as an example

Martha Kandziora, Petr Sklenář, Filip Kolář, Roswitha Schmickl

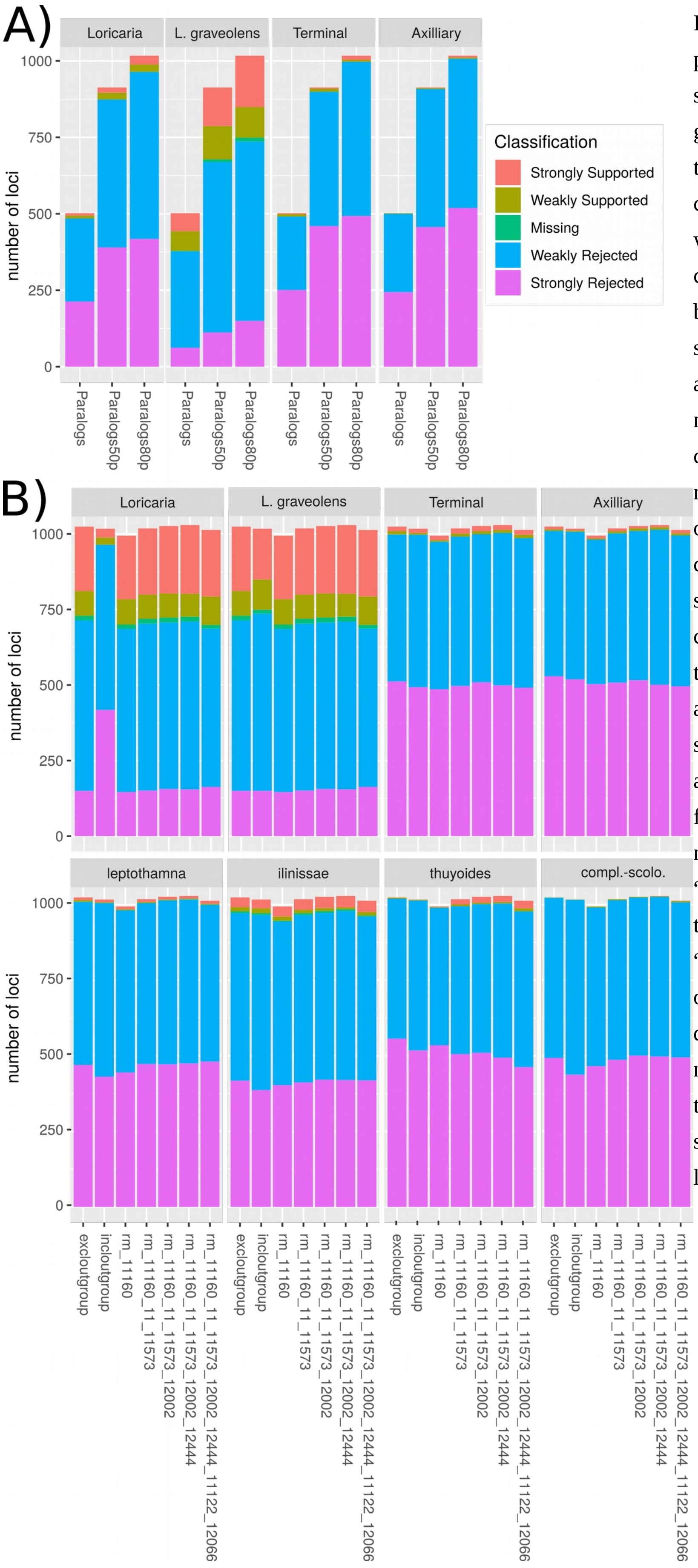

Figure S1: Discordance among gene trees for particular nodes. Results of different filtering strategies are presented. Nodes within the gene trees that are also present in the species tree and that have ≥70% support are considered as strongly supported, <70% as weakly supported. If nodes are not compatible with the species tree but would be compatible with it if branches with support <70% were collapsed, these nodes are considered as weakly rejected. Those nodes that are incompatible even after collapsing are considered as strongly rejected. A) Sample filtering for percentage of missing loci resulting in dataset 1. The column “paralogs” represents all used samples independent of sample quality. The column “Paralogs50p” represents all samples that passed the filter of 50% loci assembled, and the column “Paralogs80p” represents all samples that passed the filter of 80% loci assembled (resulting in dataset 1). B) Sample filtering for gene flow according to Dsuite resulting in dataset 2. The column “excloutgroup” represents dataset 1 without the outgroup taxa. The column “incloutgroup” represents dataset 1. The other columns represent the datasets used during the iterative Dsuite approach. The numbers provided indicate the sample IDs that were removed during the Dsuite analysis steps, as they showed introgression >5%; the last column represents dataset 2.

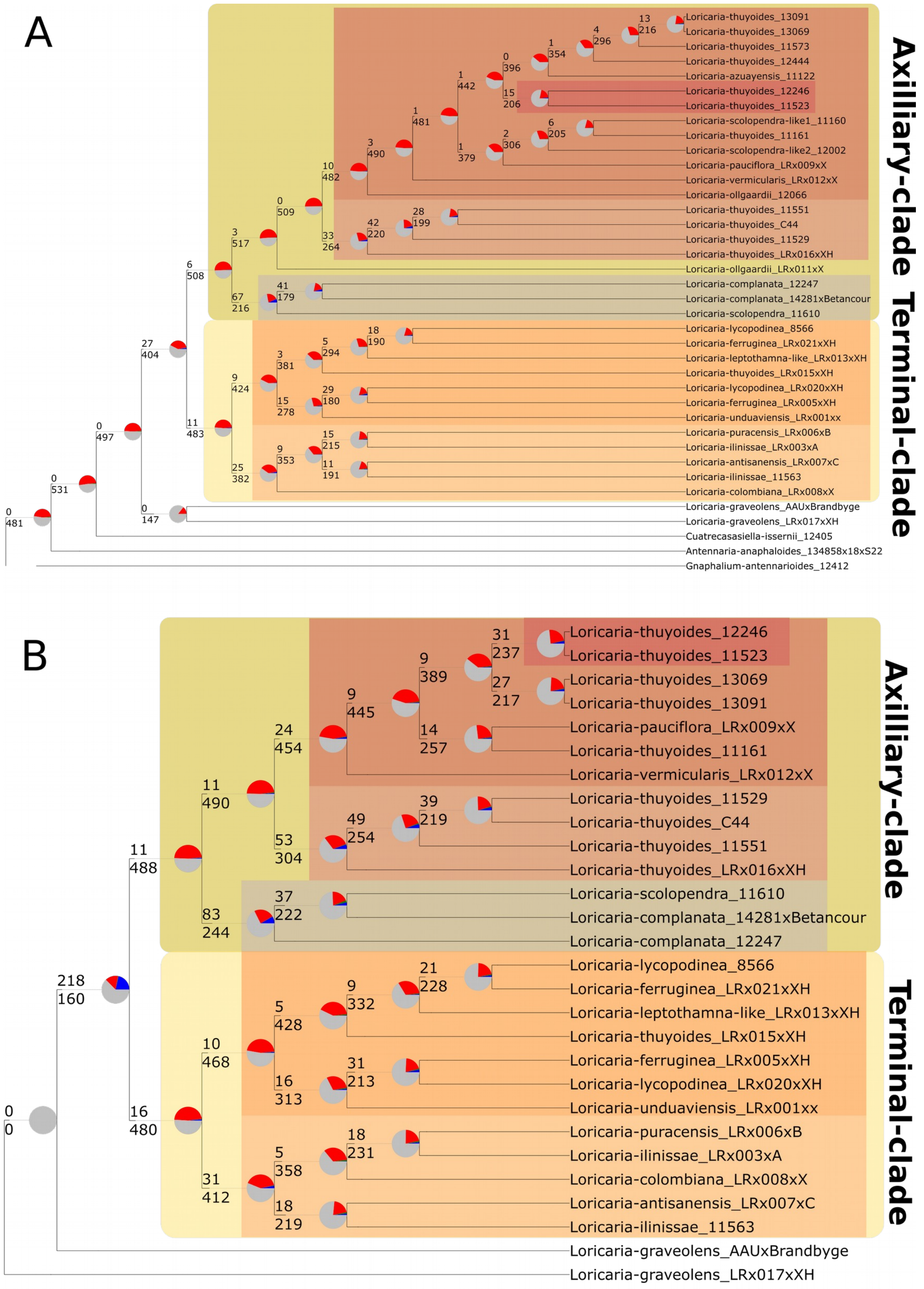

Figure S2: ASTRAL species trees with the results from the PhyParts analysis of A) dataset 1, before removing samples showing introgression according to Dsuite and B) dataset 2, after removing samples showing introgression according to Dsuite. Pie charts at nodes indicate the proportion of concordant and conflicting gene trees. Gray: uninformative gene trees (support <70% BS); blue: concordant gene trees; green: gene trees showing the most common conflicting bipartition; red: gene trees showing other conflicting bipartitions. Numbers above branches: gene trees supporting the node. Numbers below branches: gene trees not supporting the node. Colored boxes represent major clades discussed in the main text.

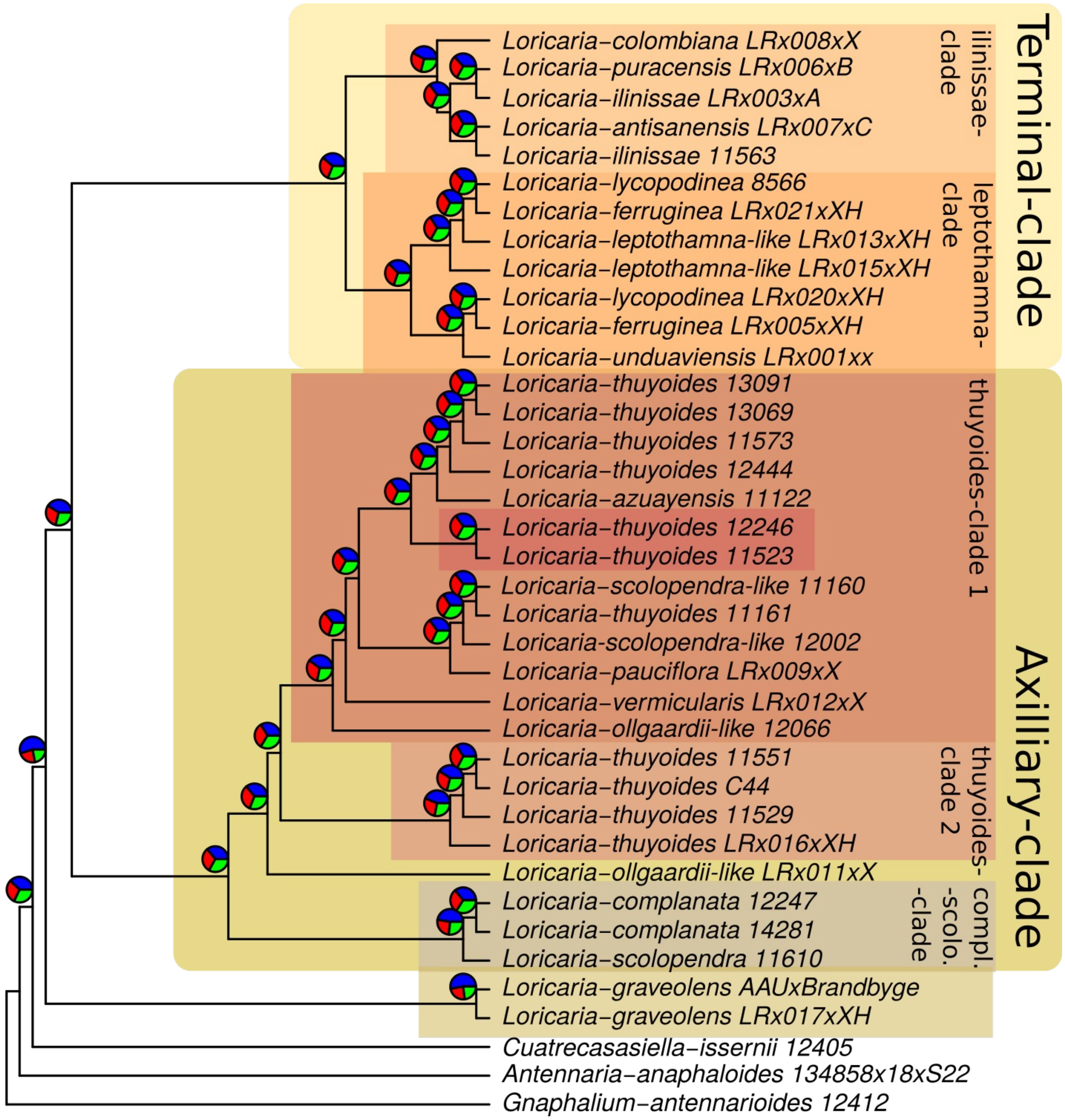

Figure S3: ASTRAL species tree with the percentage of alternative quartets for each internal branch (dataset 1). Colored boxes represent major clades discussed in the main text. Pie charts at nodes represent the percentage of alternative quartets; each color represents one alternative topology with blue being the alternative displayed as tree here.

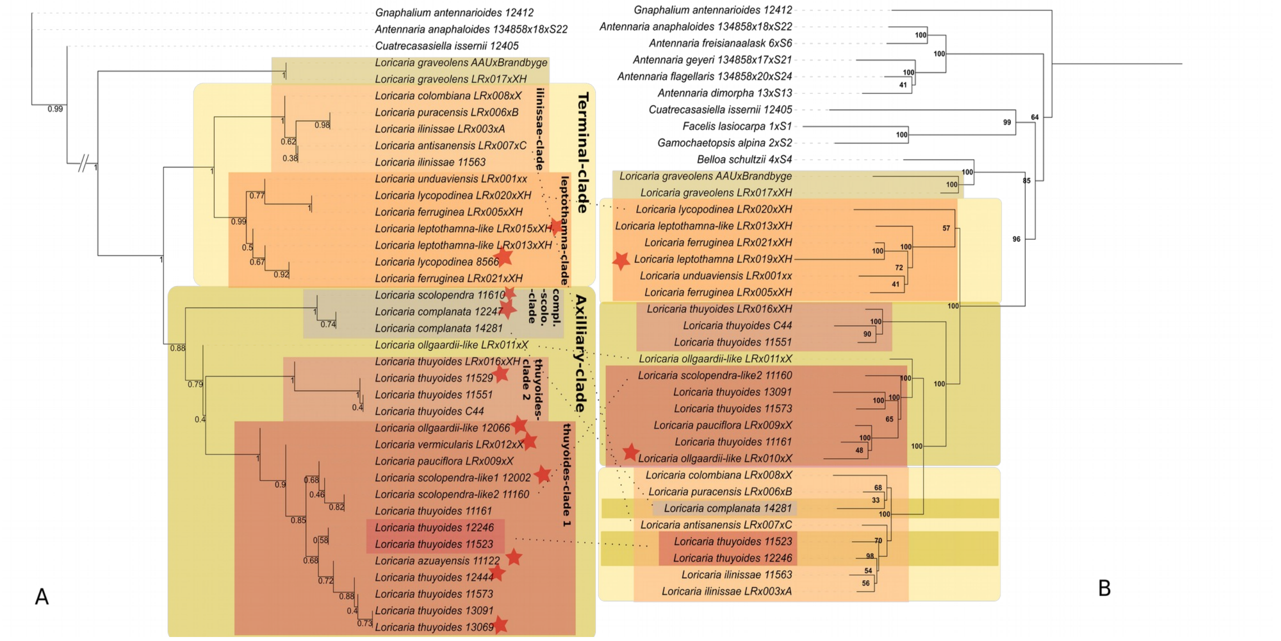

Figure S4: *Nuclear species tree of dataset 1 using ASTRAL III (A) in comparison to the concatenated tree calculated using RAxML-NG (B). Different positions of samples between clades are indicated by lines. Red stars indicate samples of* Loricaria *not present in the other phylogeny. Colored boxes represent major clades discussed in the main text. Values at nodes in A) represent local posterior probabilities and in B) bootstrap values. The branch leading to the genus has been shortened in A to increase visibility.*

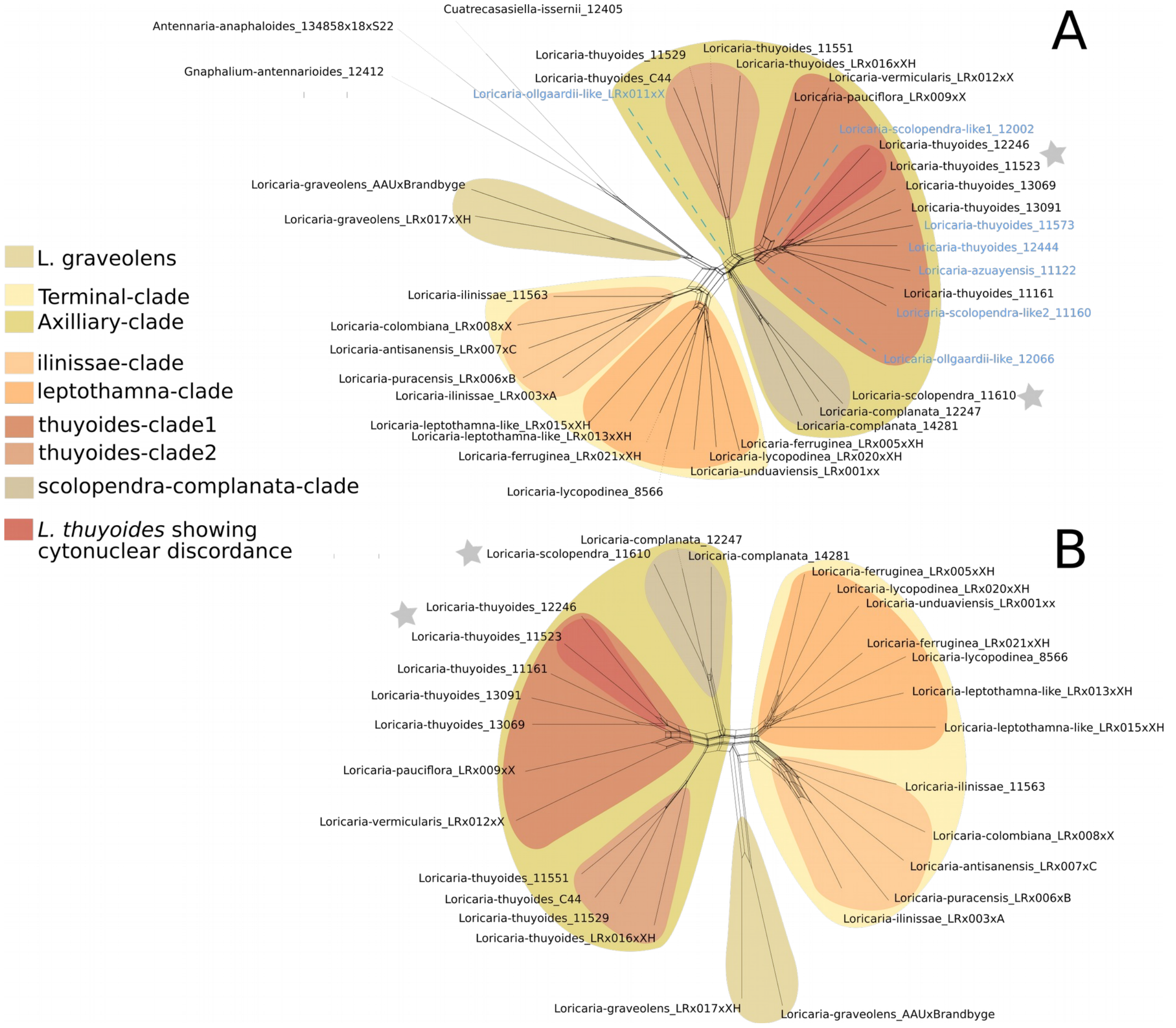

Figure S5: NeighborNet results before and after removing taxa showing introgression according to the D-statistics. A) Dataset 1: Network with all samples included after the 80% filtering. Lines leading to the outgroup are shortened. Blue dashed lines indicate samples in misplaced positions in comparison to the phylogenetic reconstruction, blue labels indicate samples that have been removed according to the Dsuite analyses . B) Dataset 2: Network after removing samples showing introgression according to the Dsuite analyses and exclusion of the outgroup. Gray stars indicate samples showing cytonuclear discordance. Colored boxes represent major clades discussed in the main text.

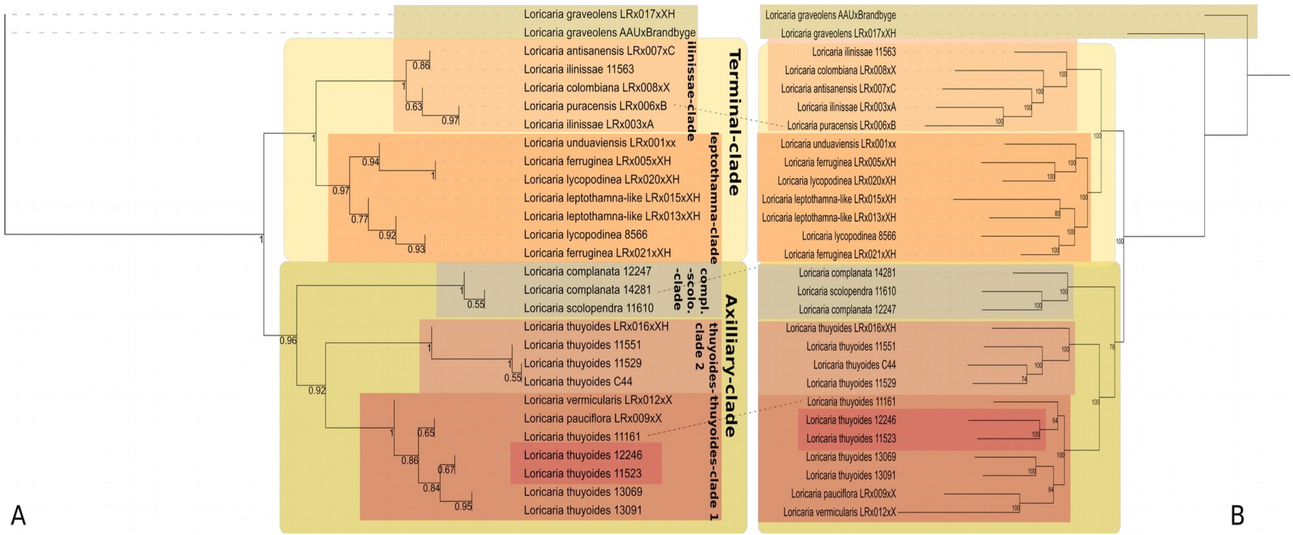

Figure S6*: Nuclear species tree of dataset 2 using ASTRAL III (A) in comparison to the concatenated tree calculated using RAxML-NG (B). Values at nodes in A) represent local posterior probabilities and in B) bootstrap values. Samples that differ in their phylogenetic placement are indicated by a line. Labeled boxes indicate major clades discussed in the main text.*

Table S1. Voucher information. Abbreviations: ECU – Ecuador, BOL – Bolivia, COL – Colombia. Herbarium codes: MA: Real Jardín Botánico (Spain, Madrid), PRC: Charles University (Czech Republic, Prague), BONN: University of Bonn (Germany. Bonn), AAU: Aarhus University (Denmark, Aarhus).

Code PRC	Genus	Species	Date collected	State	Province	Locality	Habitat	Altitude in m	Decimal Latitude	Decimal Longitude	Collector	Herbarium code	HybSeq run
12405	*Cuatrecasasiella*	*issernii*	02/11/2009	PERU	Tungurahua	Parque Nacional Llanganatis, grass paramo above the Laguna Pisayambo	Wet places in the paramo	3680	-1.105278	-78.385278	P. Sklenar	PRC	run2
12412	*Gnaphalium*	*antennarioides*	02/11/2009	PERU	Tungurahua	Parque Nacional Llanganatis, grass paramo above the Laguna Pisayambo	Grass paramo with tussocks of Calamagrostis intermedia	3910	-1.135278	-78.369444	P. Sklenar	PRC	run2
LRxx013xXH	*Loricaria*	*"leptothamna-like"*	29/08/1989	PERU	La Libertad	Travesia de Laplap (Longotea-Unamen)	Jalca	3300				BONN	run5
LRx015xXH	*Loricaria*	*"leptothamna-like"*	16/03/2001	PERU	La Libertad	Laguna Huascacocha	Jalca	3800				BONN	run5
12066	*Loricaria*	*"ollgaardii-like"*	17/06/2009	ECU	Loja	Parque Nacional Podocarpus, paramo near the summit of Cerro Toledo	Wet shrubby subparamo vegetation	3400	-4.39166667	-79.1125	P. Sklenar, J. Mackova & P. Macek	PRC	run2
LRx011xX	*Loricaria*	*"ollgaardii-like"*	11/06/2018	ECU	Loja	Parque Nacional Yacuri, above Laguna Negra, to the northern peaks of Cerros Los Picachos	Humid grass paramo with rocks	3254	-4.71277778	-79.4302778	Sklenar P., Ptacek J., Klimesova J., Klimes A.	PRC	run5
LRx010xX	*Loricaria*	*"ollgaardii-like"*	11/06/2018	ECU	Loja	Parque Nacional Yacuri, above Laguna Negra, to the northern peaks of Cerros Los Picachos	Humid grass paramo with rocks	3254	-4.71277778	-79.4302778	Sklenar P., Ptacek J., Klimesova J., Klimes A.	PRC	run5
12002	*Loricaria*	*"scolopendra-like"*	14/06/2009	ECU	Loja	Paramo de Fierro Urco, to the southwest of	Rocky summit ridge, open paramo	3670	-3.69333333	-79.3505556	P. Sklenar, J. Mackova & P.	PRC	run2

						Saraguro	vegetation.				Macek		
11160	*Loricaria*	*"scolopendra-like2"*	23/07/2008	ECU	Morona Santiago	Mountain pass of the road Gualaceo-Limon, along the way from the pass towards the antennas (Pato Cocha 3598 m), humid bamboo subparamo with scattered shrubs.		3470	-3.00327778	-78.6614444	P. Sklenar & J. Karbulkova	PRC	run2
13034	*Loricaria*	*"vermicularis"*	20/11/2010	ECU	Loja	Cordillera Cordoncillo, El Quingueado (IGM map Saraguro) along road (under construction) from Babes (ca 2 km east of Urdaneta) towards 28 de Mayo	Marshy habitats in grass paramo with Pinus plantation, disturbed by burning	3170	-3.57958333	-79.1091944	P. Sklenar & V. Zeisek	PRC	run2
LRx012xX	*Loricaria*	*"vermicularis"*	11/10/2018	ECU	Loja	Cordillera Cordoncillo, along road from Babes (ca 2 km east of Urdaneta) towards 28 de Mayo	Rocky outcrops and marshy places in grass paramo with Pinus plantation, disturbed by burning	3070	-3.5775	-79.1086111	Sklenar P., Ptacek J., Klimesova J., Klimes A.	PRC	run5
12448	*Loricaria*	*antisanensis*	11/06/2009	ECU	Napo	North-eastern side of Volcan Antisana	Shrubby lower superparamo veetation	4120	-0.45166667	-78.1255556	P. Sklenar	PRC	run2
LRx007_C	*Loricaria*	*cf. antisanensis*	11/05/2009	ECU	Napo	Northern side of Volcan Antisana, near quebrada of Rio Blanco	Low superparamo vegetation	4400	-0.455	-78.1391667	P. Sklenar	PRC	run5
LRx006_B	*Loricaria*	*cf. puracensis*	09/10/2008	COL	Cauca	Paramo de Purace, along the footpath from Pilimbala to Volcan Purace, ca 1 km above of the old military base	Open shrubby superparamo	4165	2.329388889	-76.39530556	D. Vasquez, P. Sklenar, E. Duskova et al.	PRC	run5
FAA 633	*Loricaria*	*colombiana*	10/04/2008	COL	Caldas-Tolima	Los Nevados, paramo around the crossing of	Semi-open grassy paramo with	4060	4.939194444	-75.3494722	Fabio Andres A., P. Sklenar	PRC	run2

						the road from Termales to Nevado El Ruiz with the road to Libano	scattered shrubs				et. al.		
LRx008xX	*Loricaria*	*colombiana*	02/07/2004	COL	Caldas	PNN Los Nevados, arenales en el centro de visitantes Las Brisas y el Nevado del Ruiz	Superparamo	4300	4.896497	-75.338466	Pedraza P.	PRC	run5
12335	*Loricaria*	*complanata*	24/10/2009	COL	Cauca	Paramo del Letrero, trail from Valencia towards Laguna Santiago and Laguna Suramerica	Shrubby and marshy paramo along the trail	3700	1.920611111	-76.5955833	P. Sklenar, E. Duskova, F. Kolar & D. Vasquez	PRC	run2
12346	*Loricaria*	*complanata*	26/10/2009	COL	Cundinamarca	Paramo de Chingaza, trail from Laguna Chingaza to Laguna de Media.		3640	4.505694444	-73.7330556	P. Sklenar & F. Kolar	PRC	run2
12247	*Loricaria*	*complanata*	20/10/2009	COL	Valle de Cauca	Farallones de Cali, trail from Pance to the Balcones.		3670	3.330333333	-76.6905278	P. Sklenar, E. Duskova, F. Kolar & D. Vasquez	PRC	run2
14281	*Loricaria*	*complanata*	16/07/2009	COL	Risaralda/Choco	Serrania de Tatama, PNN Tatama, Piedra Bomba	Paramo dominado por Espeletia occidentalis	3800-4200	5.162186	-76.090141	J. Betancour	PRC	run2
C56	*Loricaria*	*complanata*	13/10/2008	COL	Narino	Narino, Volcan Azufral	Marshy paramo with *Loricaria*, *Oritrophium peruvianum* and *Hypochaeris*	3892	1.09197	77.70847		PRC	run2
11521	*Loricaria*	*complanata*	16/10/2008	ECU	Carchi	Volcan Chiles, swampy area around antennas, ca 0.5 km N of the pass with the road Tufino - Maldonado	Mosaic of open stony paramo and marshes with Distichia muscoides and Plantago rigida	4160	0.806277778	-77.9422222	P. Sklenar, E. Rejzkova, F. Kolar	PRC	run2
LRx005xX	*Loricaria*	*ferruginea*	12/06/2009	PERU	La Libertad	Pr. Shore, cerca de laguna el Toro			-7.9744	-78.2339	S. Leiva, Carlos Aedo	MA	run5

H										Pérez		
LRx021xXH	*Loricaria*	*ferruginea*	28/05/1960	PERU	Acopalca	Huaytapallana	Between rocks, rare	4800			B	run5
AAU_Brandbyge	*Loricaria*	*graveolens*	25/03/1987	PERU	Puno	Abra la Raya on the Cusco/Puno border	Degraded puna vegetation (overgrazing-burning) with some low boggy parts, growing on rocky ridge	4350	-14.5	-70.983333	Brandbyge, J.	AAU	run2
LRx017xXH	*Loricaria*	*graveolens*	21/08/1960	PERU	Arequipa			4000				B	run5
11611	*Loricaria*	*ilinissae*	25/10/2008	ECU	Chimborazo	Páramos around Cerro Quilimas, along the trail Alao - Huamboya, ca 0.5 km E of the pass of the Laguna Negra	Open paramo along the path	3960	-1.77830556	-78.44375	P. Sklenar, E. Rejzkova, F. Kolar	PRC	run2
LRx003_A	*Loricaria*	*ilinissae*	16/10/2008	ECU	Carchi	Volcan Chiles, swampy area around antennas, ca 0.5 km N of the pass with the road Tufino - Maldonado	Mosaic of open stony paramo and marshes with Distichia muscoides and Plantago rigida	4160	0.806277778	-77.9422222	P. Sklenar, E. Rejzkova, F. Kolar	PRC	run5
11563	*Loricaria*	*ilinissae*	18/10/2008	ECU	Pichincha	Páramo de Iliniza, along the trail from the parking place towads the Ilinizas.	Open vegetation on sand substrate dominated by Loricaria	4295	-0.64277778	-78.6962778	P. Sklenar, E. Rejzkova, F. Kolar	PRC	run2
8679	*Loricaria*	*leptothamna*	26/08/2004	PERU	Cajamacra	Jalca de Kumulca, mountain pass on the road Cajamarca-Celendín	Humid jalca vegetation with tussock grasses	3700	-7.04	-78.25917		PRC	run2
LRx019xX	*Loricaria*	*leptothamna*	17/05/2003	PERU	Ancash	Recuay: Road Catac to Chavin de Huantar via						B	run5

H						Laguna Querococha; Abra Cahuish, western side of tunnel, 4480 m (FO 2).							
8566	*Loricaria*	*lycopodinea*	23/08/2004	PERU	La Libertad	Along road Quiruvilca-Huamachuco, between Pampas de la Julia and "Lugar arqueologico"	Dry jalca grassland with scattered rocky habitats	4050-4150	-7.98333	-78.26667		PRC	run2
LRx020xXH	*Loricaria*	*lycopodinea*	09/10/2007	Peru	Ancash	Prov. Bolognesi, road from Nuallanca to San Marcos via Lago Canrash, north of Lago Canrash,		4522	-9.7080333	-77.0306167		B	run5
LRx009xX	*Loricaria*	*pauciflora*	11/10/2018	ECU	Loja	Cordillera Cordoncillo, along road from Babes (ca 2 km east of Urdaneta) towards 28 de Mayo	Rocky outcrops and marshy places in grass paramo with Pinus plantation, disturbed by burning	3070	-3.5775	-79.1086111	Sklenar P., Ptacek J., Klimesova J., Klimes A.	PRC	run5
13035	*Loricaria*	*pauciflora*	20/11/2010	ECU	Loja	Cordillera Cordoncillo, El Quingueado (IGM map Saraguro) along road (under construction) from Babes (ca 2 km east of Urdaneta) towards 28 de Mayo	Marshy habitats in grass paramo with Pinus plantation, disturbed by burning	3170	-3.57958333	-79.1091944	P. Sklenar & V. Zeisek	PRC	run2
12319	*Loricaria*	*puracensis*	22/10/2009	COL	Cauca	Volcan Purace, the trail from the sulphur mine to the northern side of the crater.	Open superparamo vegetation	4200	2.328333333	-76.395	P. Sklenar, E. Duskova, F. Kolar & D. Vasquez	PRC	run2
11610	*Loricaria*	*scolopendra*	25/10/2008	ECU	Chimborazo	Páramos around Cerro Quilimas, along the trail Alao - Huamboya, in the pass next to the Laguna Negra	Marshy paramo strongly influenced by grazing	4010	-1.77597222	-78.4404444	P. Sklenar, E. Rejzkova, F. Kolar	PRC	run2

13108	*Loricaria*	*scolopendra*	12/03/2010	ECU	Tungurahua	Parque Nacional Llanganatis, slopes above laguna at the western side of Cerro Hermoso	Tussock grass paramo	4090	-1.22727778	-78.29875	P. Sklenar	PRC	run2
12246	*Loricaria*	*thuyoides*	20/10/2009	COL	Valle de Cauca	Farallones de Cali, trail from Pance to the Balcones.		3670	3.33033333	-76.6905278	P. Sklenar, E. Duskova, F. Kolar & D. Vasquez	PRC	run2
C44	*Loricaria*	*thuyoides*	10/09/2008	COL	Cauca	Paramo de Purace, along the footpath from Pilimbala to Volcan Purace	Paramo vegetation influenced by grazing	3650	2.357027778	-76.4028333	D. Vasquez, P. Sklenar, E. Duskova et al.	PRC	run2
11594	*Loricaria*	*thuyoides*	24/10/2008	ECU	Chimborazo	Páramo to the SW of Chimborazo, ca 2 km of the road Cruz del Arenal-San Juan.	Semi-open disturbed paramo on sandy substrate, near the rocks	4270	-1.53527778	-78.8808333	P. Sklenar, E. Rejzkova, F. Kolar	PRC	run2
11122	*Loricaria*	*azuayensis*	22/07/2008	ECU	Azuay	Superparamo vegetation to the N from the pass of the road Cuenca-Molleturo, mountain ridge towards Cerro Amarillo.		4300	-2.76961111	-79.24330556	P. Sklenar & J. Karbulkova	PRC	run2
11161	*Loricaria*	*thuyoides*	23/07/2008	ECU	Morona Santiago	Mountain pass of the road Gualaceo-Limon, along the way from the pass towards the antennas (Pato Cocha 3598 m), humid bamboo subparamo with scattered shrubs.		3470	-3.00327778	-78.6614444	P. Sklenar & J. Karbulkova	PRC	run2
11573	*Loricaria*	*thuyoides*	19/10/2008	ECU	Cotopaxi	Páramo de Quispicacha, pass between valley of Rio Pigua and Quebrada Tauricucho, ca 5 km	Open paramo vegetation on rocks strongly influenced by grazing	4440	-1.08175	-78.8421111	P. Sklenar, E. Rejzkova, F. Kolar	PRC	run2

						ESE of Chinipamba, ca 20 km SSE of Zumbagua							
13069	*Loricaria*	*thuyoides*	23/11/2010	ECU	Chimborazo	Parque Nacional Sangay, mountain ridge leading to Achipungu, above Laguna Magtayan	Superparamo vegetation with cushions, tussock grasses, and shrubs on steep rocky slope	4310	-2.23075	-78.5564167	P. Sklenar & V. Zeisek	PRC	run2
13091	*Loricaria*	*thuyoides*	24/11/2010	ECU	Chimborazo	Paramo de Chanlor, ca 16 km to the west of Guamote	Superpáramo vegetation with cushions of Azorella and Plantago rigida	4360	-1.94077778	-78.8548056	P. Sklenar & V. Zeisek	PRC	run2
11523	*Loricaria*	*thuyoides*	16/10/2008	ECU	Carchi		Grass paramo with Espeletia	3860	0.7935	-77.9104722	P. Sklenar, E. Rejzkova, F. Kolar	PRC	run2
12444	*Loricaria*	*thuyoides*	11/05/2009	ECU	Napo	Western side of Volcan Antisana, above Laguna Santa Lucia		4570	-0.46833333	-78.1633333	P. Sklenar	PRC	run2
LRx016xXH	*Loricaria*	*thuyoides*	31/10/2006	ECU	Chimborazo	Western side of Chimborazo volcano, Arenal Grande	Sparse superparamo vegetation	4200			P. Sklenar	PRC	run5
11529	*Loricaria*	*thuyoides*	16/10/2008	ECU	Sucumbíos	Páramo El Mirador, ca 6 km to the east of Huaca.	Grass paramo with Espeletia	3600	0.618555556	-77.6815	P. Sklenar, E. Rejzkova, F. Kolar	PRC	run2
11551	*Loricaria*	*thuyoides*	17/10/2008	ECU	Imbabura	Volcan Cotacachi, along the trail from the TV antennas towards the summit	Wet, semi-open grass paramo	4125	0.343666667	-78.34125	P. Sklenar, E. Rejzkova, F. Kolar	PRC	run2
LRx001xX	*Loricaria*	*unduaviensis*	2015	BOL	La Paz						P. Sklenar	PRC	run3

Table S2: Alignment information.

Species	Nuclear data			Plastome data				Clade	Sources
	Number of loci recovered	Number of paralogous sequences	Sample present in 80% of the loci assembled (dataset1)	Total number of reads	Number of plastid reads mapped	Percentage of reads mapped	Sample present in 80% of the plastome assembled (dataset1)		
Antennaria-anaphaloides_134858x18xS22	1057	270	x	2003108	34486	1.72	x	Outgroup	Mandel et al. 2019
Antennaria-dimorpha_13xS13	38	4		882192	58334	6.61	x	Outgroup	Mandel et al. 2019
Antennaria-flagellaris_134858x20xS24	977	275		1620972	27507	1.70	x	Outgroup	Mandel et al. 2019
Antennaria-freisianaalask_6xS6	193	20		590579	16208	2.74	x	Outgroup	Mandel et al. 2019
Antennaria-geyeri_134858x17xS21	891	229		1174674	14336	1.22	x	Outgroup	Mandel et al. 2019
Antennaria_linperu_S13	2	0		515260	12477	2.42		Outgroup	Mandel et al. 2019
Antennaria-pulcherrima_S1	808	158		446245	7921	1.78		Outgroup	Mandel et al. 2019
Belloa-schultzii_4xS4	257	28		469218	9938	2.12	x	Outgroup	Mandel et al. 2019
Cuatrecasasiella-issernii_12405	1122	287	x	3372973	43079	1.27	x	Outgroup	SAMN22857823
Facelis-lasiocarpa_1xS1	742	133		851693	98015	11.5	x	Outgroup	Mandel et al. 2019
Gamochaetopsis-alpina_2xS2	449	66		610912	53756	8.80	x	Outgroup	Mandel et al. 2019
Gnaphalium-antennarioides_12412	1121	266	x	3425011	44350	1.29	x	Outgroup	SAMN22857824
Luciliocline-subspicata_3xS3	574	89		189284	3769	1.99		Outgroup	Mandel et al. 2019
Loricaria-antisanensis_12448	593	87		1531191	14583	0.95		Loricaria	SAMN22857834
Loricaria-antisanensis_LRx007xC	1017	207	x	1251133	13066	1.04	x	Loricaria	SAMN22857835
Loricaria-colombiana_FAA633	481	60		1367653	11144	0.81		Loricaria	SAMN22857837
Loricaria-colombiana_LRx008xX	1062	216	x	1209735	6415	0.53	x	Loricaria	SAMN22857838
Loricaria-complanata_11521	601	110		2251678	11908	0.53		Loricaria	SAMN22857844
Loricaria-complanata_12247	1030	227	x	3024670	13092	0.43		Loricaria	SAMN22857841
Loricaria-complanata_12335	521	92		1152645	4427	0.38		Loricaria	SAMN22857839
Loricaria-complanata_12346	494	90		1925890	14158	0.74		Loricaria	SAMN22857840
Loricaria-complanata_14281	1099	245	x	2628557	10588	0.40	x	Loricaria	SAMN22857842
Loricaria-complanata_C56	871	184		2113378	9859	0.47		Loricaria	SAMN22857843
Loricaria-ferruginea_LRx005xXH	1162	305	x	9613212	58148	0.60	x	Loricaria	SAMN22857845
Loricaria-ferruginea_LRx021xXH	1163	292	x	9859463	100975	1.02	x	Loricaria	SAMN22857846
Loricaria-graveolens_AAUxBrandbyge	1112	256	x	3570556	11575	0.32	x	Loricaria	SAMN22857847
Loricaria-graveolens_LRx017xXH	1144	272	x	12026054	154771	1.28	x	Loricaria	SAMN22857848
Loricaria-ilinissae_11563	1126	256	x	3470625	53308	1.54	x	Loricaria	SAMN22857851
Loricaria-ilinissae_11611	379	63		1737466	11253	0.65		Loricaria	SAMN22857849
Loricaria-ilinissae_LRx003xA	1035	212	x	1162074	10214	0.88	x	Loricaria	SAMN22857849
Loricaria-leptothamna_8679	760	145		2187655	8689	0.40		Loricaria	SAMN22857852

Loricaria-leptothamna_LRx019xXH	859	152		7490554	14865	0.20	x	Loricaria	SAMN22857853
Loricaria-lycopodinea_8566	1084	249	x	3076195	6073	0.20		Loricaria	SAMN22857854
Loricaria-lycopodinea_LRx020xXH	1163	279	x	8373723	23474	0.28	x	Loricaria	SAMN22857855
Loricaria-leptothamna-like_LRx013xXH	1105	247	x	1591611	9233	0.58	x	Loricaria	SAMN22857825
Loricaria-leptothamna-like_LRx015xXH	1075	234	x	1394577	2836	0.20		Loricaria	SAMN22857826
Loricaria-ollgaardii-like_12066	1104	250	x	3298756	16261	0.49		Loricaria	SAMN22857827
Loricaria-ollgaardii_LRx010xX	923	185		921687	5027	0.55	x	Loricaria	SAMN22857829
Loricaria-ollgaardii-like_LRx011xX	1071	236	x	1420201	12581	0.89	x	Loricaria	SAMN22857828
Loricaria-pauciflora_13035	935	197	x	2342712	9865	0.42		Loricaria	SAMN22857857
Loricaria-pauciflora_LRx009xX	1046	220		1540569	16296	1.10	x	Loricaria	SAMN22857856
Loricaria-puracensis_12319	545	90		1733320	17606	1.06		Loricaria	SAMN22857858
Loricaria-puracensis_LRx006xB	1039	218	x	1437326	15534	1.08	x	Loricaria	SAMN22857836
Loricaria-scolopendra_11610	1087	245	x	2971769	14026	0.47		Loricaria	SAMN22857859
Loricaria-scolopendra_13108	941	202		2750271	8541	0.31		Loricaria	SAMN22857860
Loricaria-scolopendra-like_11160	1113	259	x	3223359	23262	0.72	x	Loricaria	SAMN22857831
Loricaria-scolopendra-like_12002	1033	223	x	2677502	12794	0.48		Loricaria	SAMN22857830
Loricaria-thuyoides_11161	1092	238	x	2752589	15533	0.56	x	Loricaria	SAMN22857834
Loricaria-thuyoides_11523	1078	236	x	2478310	18052	0.73	x	Loricaria	SAMN22857869
Loricaria-thuyoides_11529	1112	258	x	3030362	10315	0.34		Loricaria	SAMN22857872
Loricaria-thuyoides_11551	1104	243	x	2773436	29203	1.05	x	Loricaria	SAMN22857873
Loricaria-thuyoides_11573	1085	237	x	2398881	17267	0.72	x	Loricaria	SAMN22857866
Loricaria-thuyoides_11594	474	78		1780666	5276	0.30		Loricaria	SAMN22857863
Loricaria-thuyoides_12246	1094	244	x	2466854	26117	1.06	x	Loricaria	SAMN22857861
Loricaria-thuyoides_12444	1118	259	x	3071880	10636	0.35		Loricaria	SAMN22857870
Loricaria-thuyoides_13069	1092	245	x	2435834	11149	0.46		Loricaria	SAMN22857867
Loricaria-thuyoides_13091	1098	248	x	3155642	15657	0.50	x	Loricaria	SAMN22857868
Loricaria-thuyoides_C44	1097	245	x	2458067	12241	0.50	x	Loricaria	SAMN22857862
Loricaria-thuyoides_LRx016xXH	1147	282	x	9953388	51036	0.51	x	Loricaria	SAMN22857871
Loricaria-*azuayensis*_11122	1016	259	x	3071880	10636	0.35		Loricaria	SAMN22857864
Loricaria-unduaviensis_LRx001xX	1150	269	x	6531802	167293	2.56	x	Loricaria	SAMN22857874
Loricaria-vermicularis_13034	611	115		2103883	5531	0.26		Loricaria	SAMN22857832
Loricaria-vermicularis_LRx012xX	998	206	x	1254283	4239	0.34		Loricaria	SAMN22857833

Appendix Table S3: Summary of steps conducted during the workflow to disentangle phylogenetic discordance using ParalogWizard and HybPhyloMaker.

script	Process
PW 1a	Mapping raw reads and assembling contigs
PW 1b	Matching contigs to reference
PW 2b	Creating customized reference target file; Setting: paralog = no
PW1a	Copying customized reference to the correct folder. Mapping raw reads and assembling contigs
PW1b	Matching contigs to target file (customized reference)
PW 1a	Mapping raw reads and assembling contigs
PW 1b	Matching contigs to target file
PW 2a	Calculating sequence divergence between paralogous copies; Setting: paralog = yes
PW 2b	Creating customized reference to include paralogs; Setting: paralog = yes
PW 3	Separating exonic contigs into paralogous pairs and build alignments
HPM 4a3	Removing gaps in alignments
HPM 5	Removing missing data from alignments
HPM 6	Calculating gene trees from alignments
HPM 7	Rooting gene trees
(HPM 10)	Collapsing gene trees
HPM 8	Calculating species tree

Appendix Table S4: PhyloNet results for different maximum numbers of reticulation events, showing the five best networks per analysis and the corresponding AIC values.

Maximum number of reticulations	lnL	Number of branches	AIC	deltaAIC
0	-1697000.45	7	3394014.89	1657.80
	-1697094.53	6	3394201.06	1843.98
	-1697151.54	6	3394315.07	1957.99
	-1698278.21	7	3396570.43	4213.34
	-1698360.08	7	3396734.15	4377.07
1	-1696222.70	9	3392465.40	108.32
	-1696222.90	12	3392471.81	114.72
	-1696223.67	9	3392467.34	110.25
	-1696501.69	10	3393025.38	668.29
	-1696505.75	10	3393033.50	676.41
2	-1696175.64	11	3392377.28	20.19
	-1696186.16	12	3392400.32	43.24
	-1696218.50	12	3392465.00	107.91
	-1696219.24	10	3392462.47	105.39
	-1696222.13	13	3392474.25	117.17
**3**	**-1696163.54**	**12**	**3392357.09**	**0.00**
	-1696179.81	15	3392395.61	38.53
	-1696186.48	13	3392404.95	47.87
	-1696219.21	9	3392462.42	105.34
	-1696322.78	11	3392673.55	316.47
4	-1696166.84	18	3392377.67	20.59
	-1696177.51	14	3392391.01	33.93
	-1696181.33	17	3392404.65	47.57
	-1696183.69	11	3392397.38	40.30
	-1696221.89	11	3392473.78	116.69
5	-1696169.47	13	3392374.94	17.86
	-1696170.50	17	3392385.00	27.91
	-1696184.73	12	3392403.47	46.38
	-1696221.47	10	3392472.95	115.86
	-1696221.95	12	3392477.90	120.81
